# asmbPLS: biomarker identification and patient survival prediction with multi-omics data

**DOI:** 10.3389/fgene.2024.1444054

**Published:** 2024-11-22

**Authors:** Runzhi Zhang, Susmita Datta

**Affiliations:** Department of Biostatistics, University of Florida, Gainesville, FL, United States

**Keywords:** feature selection, mbPLS, multi-omics, PLS, prediction, survival

## Abstract

**Introduction:**

With the advancement of high-throughput studies, an increasing wealth of high-dimensional multi-omics data is being collected from the same patient cohort. However, leveraging this multi-omics data to predict survival outcomes poses a significant challenge due to its complex structure.

**Methods:**

In this article, we present a novel approach, the Adaptive Sparse Multi-Block Partial Least Squares (asmbPLS) Regression model, which introduces a dynamic assignment of penalty factors to distinct blocks within various PLS components, facilitating effective feature selection and prediction.

**Results:**

We compared the proposed method with several state-of-the-art algorithms encompassing prediction performance, feature selection and computation efficiency. We conducted comprehensive evaluations using both simulated data with various scenarios and a real dataset from the melanoma patients to validate the effectiveness and efficiency of the asmbPLS method. Additionally, we applied the lung squamous cell carcinoma (LUSC) dataset from The Cancer Genome Atlas (TCGA) to further assess the feature selection capability of asmbPLS.

**Discussion:**

The inherent nature of asmbPLS imparts it with higher sensitivity in feature selection compared to other methods. Furthermore, an R package called asmbPLS implementing this method is made publicly available.

## 1 Introduction

The high-throughput technology has experienced a tremendous improvement, yielding the rapid and cost-effective generation of extensive omics data. This extensive data spans multiple platforms, such as genomics, transcriptomics, epigenomics, proteomics, microbiomics, and metabolomics (Hasin et al., 2017). This collectively enriches our understanding of the molecular mechanism behind different diseases. For instance, molecular phenotyping utilizing genomics and epigenomics data is poised to facilitate timely and accurate disease diagnosis and prediction, thereby enhancing the accuracy of prognostic assessments and the discernment of disease progression (Bell, 2004). Numerous studies have consistently demonstrated that genomic risks play a significant role in the development and progression and finally the patient survival of various types of diseases. Genomic factors can contribute substantially to a range of health conditions including but not limited to Alzheimer’s disease (Kamboh, 2022), congenital heart disease (Morton et al., 2022), and even certain types of cancers (Duijf et al., 2019; Wadowska et al., 2020; Haffner et al., 2021). In addition, microbiome-derived metabolites have been identified as biomarkers contributing to a wide range of diseases such as inflammatory bowel disease (Wlodarska et al., 2017), colorectal cancer (Louis et al., 2014), type II diabetes (Patterson et al., 2016), asthma (Lee-Sarwar et al., 2020; Chen and Blaser, 2007), as well as obesity (Ejtahed et al., 2020).

In the past decade, many bioinformatics tools have been developed to enable the analysis of individual omics data (Blekherman et al., 2011; Roumpeka et al., 2017; Calderón-González et al., 2016). As high-throughput studies advance, acquiring multiple types of omics data for the same patient becomes achievable. Hence, researchers have increasingly shifted their focus from single-omics analysis to multi-omics analysis. In multi-omics analysis, datasets are organized into blocks, with each block representing variables from a particular type of omics data observed across a cohort of individuals. Usually, multi-omics data has several important characteristics: 1) high dimensionality: the number of features typically vastly outnumbers the sample size; 2) sparsity: some features are detectable only in a minority of samples; 3) Collinearity: the features across different blocks are not independent. In addition, variables from different blocks can have different numbers of features and their structures. Therefore, integrating diverse omics datasets from various platforms and technologies while ensuring data quality is a complex yet an essential task. Integrating multi-omics data is particularly significant, as each type of omics data can offer distinct insights, allowing for a comprehensive and systematic understanding of the complex relationship between the omics data and the host. Moreover, leveraging multi-omics data provides an opportunity to develop prediction models with high accuracy, empowering us to make informed predictions about various phenotypic outcomes, such as patients’ survival time (Yang et al., 2022; Ribeiro et al., 2022; Lin et al., 2022). Predicting patient survival using multi-omics data can greatly aid healthcare professionals in crafting precise treatment strategies, thus becoming a cornerstone of personalized medicine. By pinpointing patients at higher risk of unfavorable outcomes, clinicians can customize interventions and therapies to enhance patient survival rates. This tailored approach maximizes the efficacy of treatments, ultimately improving patient care and prognosis. Furthermore, when dealing with a substantial number of features in each block, deciphering their collective contribution to the process can prove challenging. Thus, performing feature selection and dimension reduction becomes essential to pinpoint the most pertinent and predictive features within each block. This approach streamlines analysis, enhancing the interpretability and effectiveness of multi-omics data in predicting outcomes accurately.

To date, there exists a wealth of literature for dimension reduction and feature selection methods and some of them were successfully applied to this context. For example, suitable feature selection methods include Sparse Group Lasso (SGL) (Simon et al., 2013), which incorporates a convex combination of the standard Lasso penalty and the group-Lasso penalty (Yuan and Lin, 2006). Integrative Lasso with Penalty Factors (IPF-Lasso), an extension of standard Lasso, accounts for group structure by assigning different penalty factors to different blocks for feature selection and prediction (Boulesteix et al., 2017). Priority-Lasso (Klau et al., 2018) is another Lasso-based method that incorporates different groups of variables by defining a priority order for them. Additionally, Random Forest (Breiman, 2001), a powerful prediction algorithm, is known for capturing complex dependency patterns between predictors and outcome. An extension of Random Forest, called Block Forest (Hornung and Wright, 2019), has been developed for the consideration of the group structure in the data.

**FIGURE 1:**
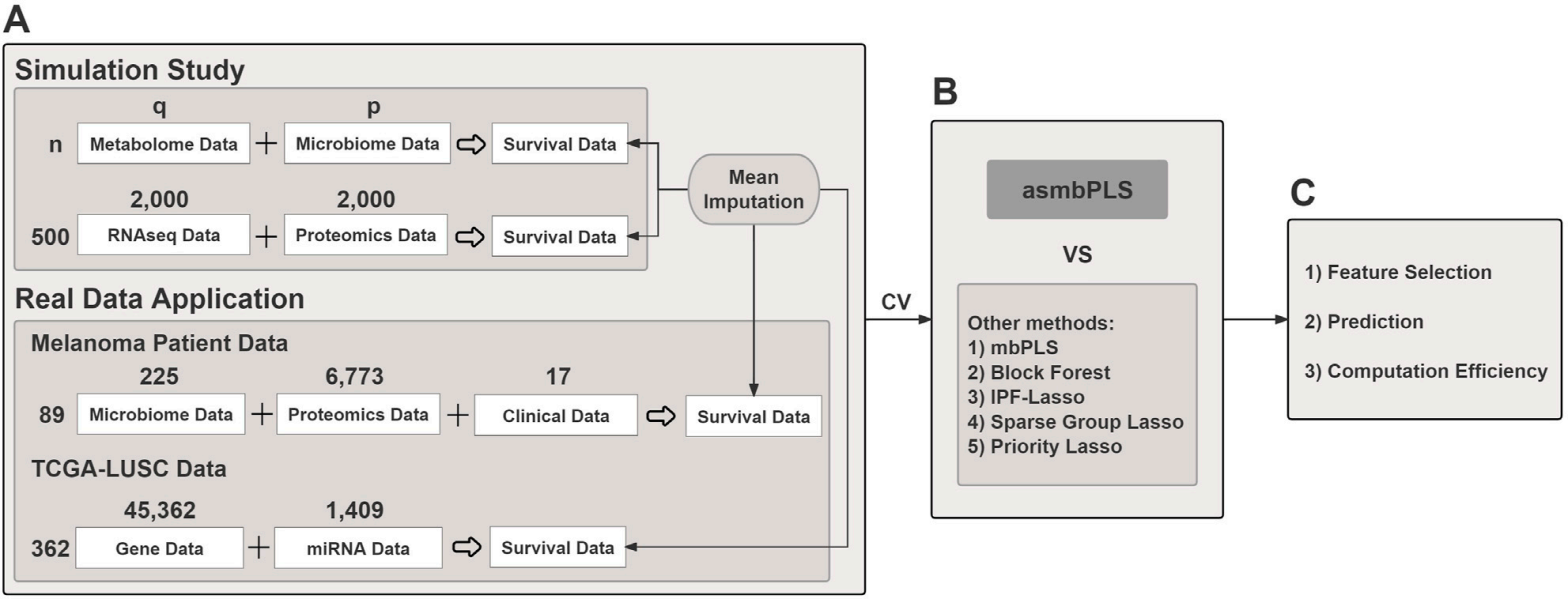
The overview of the study. **(A)** Both simulated and real data are used; **(B)** Cross validation is used for parameter tuning for different models. asmbPLS models and other competitive models with the optimal parameters settings are fitted; **(C)** Results comparison in terms of feature selection, prediction, and computation efficiency.

On the other hand, Partial least squares (PLS) regression (Geladi and Kowalski, 1986) has been used for dimension reduction and prediction using high-dimensional data. Unlike traditional linear regression, PLS is a statistical technique crafted to address scenarios featuring high-dimensional and correlated predictors within regression models. It accomplishes this by constructing latent variables, which are linear combinations of the original predictors. These latent variables aim to capture the maximum covariances between the predictors and the response variable, thus facilitating more effective modeling despite high dimensionality and correlation among predictors. Multi-block Partial Least Squares (mbPLS) (Wangen and Kowalski, 1989; Wold, 1987), initially developed for chemical systems, can be adapted for outcome prediction using multi-omics data due to the shared group structure between the multi-omics data. Furthermore, sparse mbPLS (smbPLS) (Li et al., 2012) has been developed to apply in the bioinformatics field due to the sparse nature of the data. However, smbPLS algorithm utilizes fixed penalty factors for different blocks regardless of the PLS components. This may not guarantee the optimal prediction performance. In addition, an inappropriate penalty factor may lead to the exclusion of important features from some specific blocks. This limitation hampers both biomarker identification and outcome prediction. Driven by these considerations, we introduce a novel multi-omics prediction model called adaptive sparse multi-block partial least squares (asmbPLS). This innovative framework involves the assignment of distinct penalty factors to the blocks across different PLS components by utilizing the specific quantile of feature weights as the penalty factor. This approach guarantees consistent access to insights regarding the relative significance of features within each respective block. The objective of the proposed method is to identify the subset of features most strongly associated with the outcome and subsequently predict the outcome using these selected features. Moreover, employing the quantile of feature weights as the penalty factor will enhance the interpretability of the results, making them more straightforward to understand and apply in practice. Several methodologies have emerged for predicting phenotypic features like patient survival and the onset time of severe illness. Many of these methods leverage the Cox model (Team RC, 2013; Chung and Kang, 2019; Spencer et al., 2021), whose efficacy hinges on the proportional hazard assumption. When the proportional hazard assumption is breached, an alternative to the Cox model is the accelerated failure time (AFT) model. In the AFT model, if T represents the time to a specific event, one can fit a linear regression to model a transformed variable $Y=\log\left(T\right)$ on a set of covariates $X_{1},\ldots ,X_{p}$ . Where p≫n. In this paper, we implemented asmbPLS regression method to predict the log-transformed survival time. To address censoring in the survival outcome, we utilized mean imputation (Datta, 2005) for the right-censored data. The validity of this method is confirmed through rigorous testing on both simulated and real data. The study’s framework is illustrated in Figure 1. Importantly, this method can be seamlessly applied to various types of omics data, with preprocessing tailored to the specific characteristics of each data type such as normalization. It's worth highlighting that the outcome variable employed in this method is not restricted solely to survival time; rather, it can accommodate any continuous outcome variable. An R package named asmbPLS, which implements this method, has been made publicly accessible on GitHub (https://github.com/RunzhiZ/asmbPLS).

## 2 Materials and methods

### 2.1 Right censored data imputation

In survival analysis, censoring occurs when the time-to-event information for a subject is incomplete. Among various types of censoring, right-censoring is the most prevalent. For instance, right-censoring occurs when a patient is lost to follow-up before experiencing the event of interest. Let $T_{i}^{t}$ and $T_{i}^{c}$ denote the true survival time and censored time for i th subject ( i = 1, …, n ), respectively. Then the observed survival time and the event indicator are defined as $T_{i}=\min\left(T_{i}^{t},T_{i}^{c}\right)$ and $\delta_{i}=I\left(T_{i}^{t}\leq T_{i}^{c}\right)$ , respectively. And observed data for i th subject can be presented by $\left(T_{i},\delta_{i}\right)$ , where subjects with $\delta_{i}=1$ and with $\delta_{i}=0$ are called observed and unobserved subjects, respectively. In the AFT model, we consider fitting asmbPLS regression model of $Y_{i}=\log\left(T_{i}\right)$ on $X_{i1}^{1},\ldots ,X_{ip}^{b}$ , where $X_{ip}^{b}$ is the *p*th feature of a specific omics data in block *b* for *i*th sample. Since the $T_{i}^{t}$ are not available for the individuals with $\delta_{i}=0$ , the general prediction method will not apply. Furthermore, disregarding censored subjects can introduce bias into predictions, while reducing the sample size leads to a loss of statistical power. To handle this, we propose to replace $Y_{i}$ by ${\tilde{Y}}_{i}$ such that (i) it has approximately the same mean function as $Y_{i}$ and (ii) it is computable from the observed data. In this study, mean imputation (Datta, 2005) is used to this end.

Under this scheme, we keep observed $Y_{i}$ intact but replace unobserved $Y_{i}$ by its expected value $Y_{i}^{*}$ given that the true survival time $T_{i}^{t}$ was larger than the censoring time $T_{i}^{c}$ . Let $S\left(t\right)$ denotes the survival function, the $Y_{i}^{*}$ can be estimated from the Kaplan-Meier curve of the survival function: $Y_{i}^{*}=\frac{\sum_{t>T_{i}^{c}}\log\left(t\right)\Delta \hat{S}\left(t\right)}{\hat{S}\left(T_{i}^{c}\right)}$ , where $\hat{S}$ is the Kaplan-Meier estimator of the survival time, and $\Delta \hat{S}\left(t\right)$ is the jump size of $\hat{S}$ at time t. Note that we need to treat the largest observation as a true failure for this calculation since it is necessary to make a tail correction if the largest observation $t_{\max}$ corresponds to a censored event. In summary, we let ${\tilde{Y}}_{i}=Y_{i}$ for observed survival time, and ${\tilde{Y}}_{i}=Y_{i}^{*}$ for unobserved survival time for the *i*th sample instead.

### 2.2 Adaptive sparse multi-block PLS algorithm

In mbPLS regression, the input matrix including B blocks (different omics data blocks) $X=\left[X^{1},\ldots ,X^{b},\ldots ,X^{B}\right]$ and block Y be the predictor matrix and outcome vector respectively on the same *n* samples. mbPLS regression reduces the dimension of each block by forming partial least squares components and regressing on those components. For the *j*th PLS (*j* = 1, 2, …) component, dimension reduction is implemented by taking a linear combination of the variables to obtain the block score $t_{bj}=\frac{X^{b}\omega_{j}^{b}}{{\left(m_{X^{b}}\right)}^{\frac{1}{2}}}$ ( b=1,…,B ) for each block, where $\omega_{j}^{b}$ represents the weights for the $X^{b}$ block variables, indicating how much each variable contributes to the construction of the latent variable $t_{bj}$ , which is obtained via the algorithm. Here, $m_{b}$ is the number of variables in $X^{b}$ block used for block scaling. After calculating $t_{j}^{b}$ for each block, we combine all block scores in a new matrix $T_{j}=\left[t_{j}^{1}\ldots t_{j}^{b}\ldots t_{j}^{B}\right]$ , which includes information from different blocks. Then, dimension reduction is conducted again by taking a linear combination of the different block scores to obtain the super score $t_{j}^{super}=T_{j}\omega_{j}^{super}$ , where $\omega_{j}^{super}$ is obtained from algorithm and represents the weights for each block. Similarly, $u_{j}$ is a summary vector of Y , i.e. $u_{j}=Yq_{j}$ ( $q_{j}$ being the Y weight for *j*th PLS component). The goal of the mbPLS algorithm is to find the parameters that maximize the covariance between two summary vectors, i.e. $t_{j}^{super}$ and $u_{j}$ . Therefore, the problem is formally expressed as follows:

max cov( $t_{j}^{super}$ , $u_{j}$ ) with $t_{bj}=\frac{X^{b}\omega_{j}^{b}}{{\left(m_{b}\right)}^{\frac{1}{2}}}$ , $T_{j}=\left[t_{j}^{1}\ldots t_{j}^{b}\ldots t_{j}^{B}\right],t_{j}^{super}=T_{j}\omega_{j}^{super}$ , and $u_{j}=Yq_{j}$ , subject to $\left|\left|\omega_{j}^{b}\right|\right|=\left|\left|\omega_{j}^{super}\right|\right|=1$ .

Once all the parameters are estimated for the first PLS component, *X* and *Y* are deflated and then the deflated *X* and *Y* are used for the calculation of the second PLS component and so on.

Furthermore, smbPLS (Li et al., 2012), a sparse version of the mbPLS, can be achieved by adding an $L_{1}$ penalty on each weight vector $\omega_{j}^{b}$ . In other words, the feature selection procedure is implemented on each $\omega_{j}^{b}$ , and the weights of unimportant variables will shrink to zero. In smbPLS algorithm, penalty factor $\lambda^{b}$ is fixed for all the PLS components in block b .

Unlike smbPLS, asmbPLS allows different penalty factors $\lambda_{j}^{b}$ for j th PLS component of block b by selecting the specific quantile of block weights as the penalty factor. Specifically, each step of the asmbPLS algorithm is listed in Table 1. In this algorithm, quantile is the function to obtain the corresponding quantile of the absolute weight, and sparse is the soft thresholding function $sparse\left(x,\lambda \right)=sign\left(x\right){\left(\left|x\right|-\lambda \right)}_{+}$ that is used to optimize the objective function with lasso penalties. Using the quantile function, we can always find the reasonable $\lambda_{j}^{b}$ that helps us to retain the most relevant variables in a data driven manner. By integrating these two functions, our objective is to determine the optimal quantile for each Partial Least Squares (PLS) component within each block, thereby maximizing the predictive performance of the proposed method. This approach not only enhances prediction accuracy but also simplifies the interpretation of the retained variables. After the model fitting, the parameters $\omega_{j}^{b}$ , $\omega_{j}^{super}$ , and $q_{j}$ could be saved for the prediction when we have the new data $X^{new}$ . Specifically, $\omega_{j}^{b}$ is used to calculate the corresponding $t_{j}^{b}$ for each block in the new data. And then $\omega_{j}^{super}$ is used for calculating the $t_{j}^{super}$ with these calculated $t_{j}^{b}$ . After that, $t_{j}^{super}{q_{j}}^{⊤}$ is calculated, which could be used as our prediction based on the first PLS component. If we want to use more PLS components, $p_{j}^{b}$ is calculated for obtaining the deflated $X^{new}$ , which could be further used for calculating $t_{j}^{super}{q_{j}}^{⊤}$ for the second PLS component, and so on. After the $t_{j}^{super}{q_{j}}^{⊤}$ for all the PLS components are calculated, they can be combined for obtaining the predicted Y . Note that the same mean and standard deviation derived from our data scaling on X and Y will also be used for scaling the new X and predicting Y .

**TABLE 1:** Pseudocode for the asmbPLS algorithm.

(1) Transform, center, and scale data $X=\left[X^{1},\ldots ,X^{b},\ldots ,X^{B}\right]$ and *Y*.
(2) For *j*th PLS component (*j* = 1, 2, …),
2.1 Take $u_{j}=Y$
2.2 Loop until convergency of $t_{j}^{super}$
2.2.1 ${\;\omega }_{j}^{b}=\frac{{X^{b}}^{⊤}u_{j}}{{u_{j}}^{T}u_{j}}$ (obtain $X^{b}$ block variable weight)
2.2.2 $\lambda_{j}^{b}=quantile\left(\left|\omega_{j}^{b}\right|,percent\right)$ (obtain penalty factor for block b )
2.2.3 $\omega_{j}^{b}=sparse\left(\omega_{j}^{b},\lambda_{j}^{b}\right)$ (variable selection based on soft thresholding function)
2.2.4 Normalize $\omega_{j}^{b}$ to $\left|\left|\omega_{j}^{b}\right|\right|=1$ (block variable weight normalization)
2.2.5 $t_{j}^{b}=\frac{X^{b}\omega_{j}^{b}}{{\left(m_{b}\right)}^{\frac{1}{2}}}$ (obtain $X^{b}$ block score)
2.2.6 $T_{j}=\left[t_{j}^{1},\ldots ,t_{j}^{b},\ldots ,t_{j}^{B}\right]$ (combine all scores in $T_{j}$ )
2.2.7 $\omega_{j}^{super}=\frac{{T_{j}}^{⊤}u_{j}}{{u_{j}}^{T}u_{j}}$ (obtain X super weight)
2.2.8 Normalize $\omega_{j}^{super}$ to $\left|\left|\omega_{j}^{super}\right|\right|$ = 1 (super weight normalization)
2.2.9 $t_{j}^{super}=\frac{T_{j}\omega_{j}^{super}}{{\omega_{j}^{super}}^{⊤}\omega_{j}^{super}}$ (obtain X super score)
2.2.10 $q_{j}=\frac{Y^{⊤}t_{j}^{super}}{{t_{j}^{super}}^{⊤}t_{j}^{super}}$ (obtain Y weight)
2.2.11 $u_{j}=\frac{Yq_{j}}{{q_{j}}^{⊤}q_{j}}$ (obtain Y score)
End
2.3 Deflation
2.3.1 $p_{j}^{b}=\frac{{X^{b}}^{⊤}t_{j}^{super}}{{t_{j}^{super}}^{⊤}t_{j}^{super}}$
2.3.2 $X^{b}=X^{b}-t_{j}^{super}{p_{j}^{b}}^{⊤}$
2.3.3 $Y=Y-t_{j}^{super}{q_{j}}^{⊤}$

### 2.3 Parameter tuning and model selection

We let the block size B = 2 and the number of PLS components = 5 here for instance. The cross validation (CV) is used to tune the quantile combination for different PLS components in different blocks. Tuning these parameters is equivalent to choosing the “degree of sparsity”, i.e. the number of non-zero weights for each PLS component in each block. The chosen quantile combination is the one resulted the best prediction. The CV procedure is presented in Table 2.

**TABLE 2:** CV procedure for the asmbPLS algorithm.

(1) Randomly place the samples into K roughly equal groups. And we assume that the ratios of observed samples to unobserved samples are the same in all the groups. Since we are only interested in the observed samples, the observed samples in each group will be serving as the validation set in turn with the samples from all the other groups serving as the training set.
(2) For k=1,…,K , for convenience, the same set of combinations of degree of sparsity, i.e. $quantile^{block_{1}}=\left\{q_{1}^{block_{1}},..,q_{a}^{block_{1}}\right\}$ and $quantile^{block_{1}}=\left\{q_{1}^{block_{2}},\ldots ,q_{b}^{block_{2}}\right\}$ are used for different PLS components.
For the number of used PLS components *l* (*l* starts from 1),
a) Fit asmbPLS models using the first *l* PLS component(s) with different quantile combinations used for the *l*th PLS component based on the training set.
b) Predict the validation set using fitted asmbPLS models.
c) Calculate the (scaled) K-fold mean squared error (MSE) for each combination:
$\text{MS}E_{K-fold,obs}=\frac{1}{\sigma^{2}K}\sum_{k=1}^{K}\frac{1}{n_{k}}\sum_{i=1}^{n_{k}}{\left({\hat{Y}}_{k,i}-Y_{k,i}\right)}^{2}$
where n_k_ is the number of samples in thekth validation set, and σ^2^ (details can be found in the section “Simulation Strategies”) is used for scaling.
d) Choose the combination with the lowest MSE for the *l*th PLS component.
e) Data deflation for *X* and *Y*, let *l* = *l* +1.
f) Repeat steps (a) – (e) until we obtain the combinations for all the 5 PLS components. For step (a), the combination with the lowest MSE obtained from the previous steps will be used for the first (*l* - 1)th PLS component(s).

The selection of folds for the CV, K and the number of quantile combinations will largely impact the computational efficiency. We choose K to be 5 in our study, which is large enough for parameter tuning. The selection of the quantiles for each block should be based on prior knowledge of the corresponding omics data. For example, assuming only a small proportion of features are relevant, then a higher quantile should be considered to retain fewer features.

With the information from the CV, we can determine the number of PLS components used for prediction also. Usually, the optimal number of PLS components is the one that corresponds to the lowest mean squared error (MSE) in CV. However, due to the over-fitting issue, we allow the selection of fewer components if the decrease of MSE is minimal when one more component is included. The strategy for selecting the number of PLS components is summarized: 1) Let the initial number of components be comp = 1; 2) Check whether including one more component decreases the MSE by 5%, i.e. $MSE_{comp+1}\leq MSE_{comp}\times 0.95$ ; 3) If so, then we go to the next PLS component (*comp)* i.e. comp = comp + 1 and back to step 2), otherwise, let comp be the selected number of components.

### 2.4 Technicalities and implementation

All the implementations were conducted using R 4.1.0 (Team RC, 2013) in the “HiperGator 3.0” high-performance computing cluster, which includes 70,320 cores with 8 GB of RAM on average for each core, at the University of Florida. We compared the proposed method, i.e. asmbPLS, with mbPLS, Block Forest, IPF-Lasso, Sparse Group Lasso, and Priority-Lasso using both the simulated and the real data. The choice of parameters for each method followed the suggestion from the corresponding R package tutorial. All these methods make use of group structure information and enable us to do the prediction.

### 2.5 Data Source

The R codes for the simulation study have been made publicly accessible on https://github.com/RunzhiZ/Runzhi_Susmita_asmbPLS_2024. The simulated ovarian cancer dataset was generated with OmicsSIMLA (Chung and Kang, 2019) (https://omicssimla.sourceforge.io/simuomicsTCGA.html), which includes RNA-seq data and proteomics data. The melanoma dataset was obtained from (Spencer et al., 2021) (https://github.com/mda-primetr/Spencer_et_al_2021), which includes progression-free survival interval/status, clinical covariates, and various types of omics data such as microbiome data and proteomics data for melanoma patients. The lung squamous cell carcinoma (LUSC) from The Cancer Genome Atlas (TCGA) was obtained via the Genomic Data Commons (GDC) data portal (https://portal.gdc.cancer.gov/). Gene and miRNA expression data, along with survival data, were downloaded and pre-processed using R package *TCGAbiolinks* (Colaprico et al., 2016).

## 3 Results

### 3.1 Simulation study 1

#### 3.1.1 Simulation strategies

We simulated n samples, q bacterial taxa and p metabolites to mimic the real microbiome and metabolome data, where the two types of omics data were simulated to be correlated. Subsequently, we generated censored survival time based on the simulated microbiome and metabolome data.

##### 3.1.1.1 Microbiome data

We simulated the microbiome data using the Dirichlet-multinomial (DM) distribution (Chen and Li, 2013) to accurately capture the over-dispersed taxon counts. We denote $Q=\left(Q_{1},Q_{2},\ldots ,Q_{q}\right)$ as the observed counts for q bacterial taxa. The most common model for count data is the multinomial model, whose probability function is given as:

$$f_{M}\left(Q_{1},Q_{2},\ldots ,Q_{q};\phi \right)=\left(\begin{array}{l} Q_{+}\\ Q \end{array}\right)\prod_{j=1}^{q}\phi_{j}^{y_{j}}$$

where $Q_{+}=\sum_{j=1}^{q}Q_{j}$ is the total taxon count, which is determined by the sequencing depth, and $\phi =\left(\phi_{1},\phi_{2},\ldots ,\phi_{q}\right)$ are the underlying taxon proportions with $\sum \phi_{j}=1$ . The sequencing depth-induced variability can lead to different $Q_{+}$ in different samples.

The DM distribution assumes that proportions ϕ used in the multinomial model come from the Dirichlet distribution (Mosimann, 1962) and the probability function is given by:

$$f_{D}\left(\phi_{1},\phi_{2},\ldots ,\phi_{q};\gamma \right)=\frac{\Gamma \left(\gamma_{+}\right)}{\prod_{j=1}^{q}\Gamma \left(\gamma_{j}\right)}\prod_{j=1}^{q}\phi_{j}^{\gamma_{j}-1}$$

where $\gamma =\left(\gamma_{1},\gamma_{2},\ldots ,\gamma_{q}\right)$ are positive parameters, generating from the uniform distribution, $\gamma_{+}=\sum_{j=1}^{q}\gamma_{j}$ and $\Gamma \left(\cdot \right)$ is the Gamma function. Consequently, the DM distribution is the result of a compound multinomial distribution with weights from the Dirichlet distribution:

$$f_{DM}\left(Q_{1},Q_{2},\ldots ,Q_{q};\gamma \right)=\int f_{M}\left(Q_{1},Q_{2},\ldots ,Q_{q};\phi \right)f_{D}\left(\phi ;\gamma \right)d\phi$$

In summary, to generate microbiome data, we first generated $\gamma ∼uniform\left(0,1\right)$ . γ was then used for generating ϕ , and the generated ϕ with the $Q_{+}∼uniform\left(m,2m\right)$ can be used to generate taxon counts for each sample, where m was used to control the abundance of microbial features in a specific sample. Therefore, a n×q microbiome data matrix $X^{\text{micro}.count}$ was generated with rows indicate the samples and columns indicate the microbial taxa. Microbial relative abundance $X^{\text{micro}.relative\;abundance}$ was then calculated from the count data, which will be used in the downstream simulation.

##### 3.1.1.2 Metabolome data

Once microbiome data was generated, it can be used to simulate the metabolome data due to the associations between microbiome data and metabolome data. Notice that $X^{\text{micro}.relative\;abundance}$ should be scaled first to control the microbial effect. Let a n×p matrix $X^{meta}$ be the simulated metabolome data, let $X_{ik}^{meta}$ be the intensity of k th metabolite in i th sample, we assumed that the metabolite level of $X_{ik}^{meta}$ was consisted of three parts:

$$X_{ik}^{meta}=\mu_{k}+E_{ik}+\epsilon_{ik}=\mu_{k}+X_{i}^{micro.scale}\beta_{k}+\epsilon_{ik}$$

where $\mu_{k}$ denoted the average intensity of metabolite k , $E_{ik}=X_{i}^{micro.scale}\beta_{k}$ was the microbial effect for metabolite k in i th sample and $\epsilon_{ik}$ was the random error term. Here, we have taken $\mu_{k}∼uniform\left(4,8\right)$ and $\epsilon_{ik}∼N\left(0,1\right)$ . $\beta^{\left(e\right)}=\left(\beta_{1},\ldots ,\beta_{k},\ldots ,\beta_{p}\right)$ was a q×p matrix to indicate the effect of q microbial taxa on p metabolites. Among the q×p pairs in the $\beta^{\left(e\right)}$ matrix, e pairs were selected randomly to have non-zero values with all the other elements in $\beta^{\left(e\right)}$ to equal to zero. And among these e pairs, half of the pairs were randomly selected to have positive values with another half to have negative values. In other words, $\left|\beta_{non\;zero}^{\left(e\right)}\right|∼uniform$ ( A , 2× A ), different values of A indicate different scales of association between microbiome and metabolome data.

##### 3.1.1.3 Censored survival time

Following the generation of the metabolome data, a similar scaling process was applied. Subsequently, the scaled metabolome data was integrated with the scaled microbiome data to facilitate the generation of survival time. Under the AFT framework, we assumed that both microbiome data and metabolome data collectively impact the actual survival time:

$$Y^{t}=\log\left(T^{t}\right)=baseline+X^{micro.scale}\beta^{micro}+X^{meta.scale}\beta^{meta}+\epsilon$$

where $baseline=\log\left(500\right)$ indicated the logarithm of the baseline survival time for subjects, $\beta^{micro}$ and $\beta^{meta}$ were the coefficients to decide the associations between the features and the survival time, ϵ was the random error term. Two types of error distributions were considered here: 1) Normal distribution, indicating the lognormal distribution for survival time; 2) logarithms of Weibull distribution, resulting in the Weibull distribution for survival time. Specifically, in both cases we set $\epsilon =\sqrt{r}\sigma Z$ , where Z is either $N\left(0,1\right)$ or $\left[\log\left\{Weibull\left(5,1\right)\right\}-E\;\log\left\{Weibull\left(5,1\right)\right\}\right]/\sqrt{Var\left[\log\left\{Weibull\left(5,1\right)\right\}\right]}$ , $\sigma^{2}=\beta^{T}\Sigma_{X}\beta$ with $\beta ={\left(\beta^{{micro}^{T}},\beta^{{meta}^{T}}\right)}^{T}$ and r was the noise to signal ratio. In addition, to simulate the censored time, the censoring variable c was taken to be $\sqrt{r}\sigma \;N\left(-\Phi^{-1}\left(censoring\;rate\right),1\right)$ , where Φ was the cumulative distribution function of the standard normal distribution. It was added to the $Y^{t}$ to form the $Y^{c}$ if c≤0 . For c > 0, the true survival time $Y^{t}$ will be used.

For β mentioned above, we considered six settings that we believe cover a broad range of situations. Details are listed in Table 3. In setting (1), only a limited number of relevant features exist within each block. In setting (2), for both blocks, the coefficients are fast decaying with only a small proportion of features contributing to the outcome. Setting (3) is like setting (2) but with a slower decay. Setting (4) corresponds to the situation where all the features have equal contributions to the outcome. In setting (5), there are different numbers of relevant features in different blocks (number of relevant taxa > number of relevant metabolites). Setting (6) is like setting (5) but with the number of relevant taxa < the number of relevant metabolites. We normalized the vector of β in each case to control the effect of features on survival and for computational stability.

**TABLE 3:** Different settings for β . j and k are the indices for microbial taxa and metabolites, respectively.

Setting for $\beta ={\left(\beta^{{micro}^{T}}\;\beta^{{meta}^{T}}\right)}^{T}$	$\beta_{j}^{micro}$ (*j* = 1,…, *p*)	$\beta_{k}^{meta}$ (*k* = 1,…, *q*)
(1)	for 1≤j≤5 , $\beta_{j}^{micro}=j$ , for j > 5 , $\beta_{j}^{micro}=0$	for 1≤k≤5 , $\beta_{k}^{meta}=k$ , for k > 5 , $\beta_{k}^{meta}=0$
(2)	$\exp\left(-j\right)$	$\exp\left(-k\right)$
(3)	$\frac{1}{j}$	$\frac{1}{k}$
(4)	1	1
(5)	for 1≤j≤10 , $\beta_{j}^{micro}=j$ mod 5 if j mod 5 < 5, and = 5 otherwise, for j > 10 , $\beta_{j}^{micro}=0$	for 1≤k≤5 , $\beta_{k}^{meta}=k$ , for k > 5 , $\beta_{k}^{meta}=0$
(6)	for 1≤j≤5 , $\beta_{j}^{micro}=j$ , for j > 5 , $\beta_{j}^{micro}=0$	for 1≤k≤10 , $\beta_{k}^{meta}=k$ mod 5 if k mod 5 < 5, and = 5 otherwise, for k > 10 , $\beta_{k}^{meta}=0$

#### 3.1.2 Simulation results

A variety of simulation settings were considered to account for different scenarios. In low dimensional setting, q and p were taken to be 200, the other parameter values used in this simulation were: m = 20,000 to control the abundance of microbial features in samples, e = 200 to indicate the number of randomly selected non-zero effects between microbial features and metabolites, A = 0.5 or 2 to denote moderate or high correlation between microbiome and metabolome data, r = 0, 0.1, 0.2, 0.5, 1 to indicate different scales of noise and censoring rate ( cr ) = 0.1, 0.3, 0.5, 0.7 to simulate different censoring rates in survival analysis. Sample size n was taken to be 100, and an additional $n_{test}$ = 100 samples were generated using the same design parameters to serve as the test set. For each scenario, we simulated 100 datasets. We also conducted simulations in mixed dimension ( q = 1,000 and p = 200, m = 100,000, e = 500) and high dimension ( q = p = 1,000, m = 100,000, e = 1,200) settings, the results are present below.

Since the microbial data were presented in the form of relative abundance, we imputed the zero value with the small pseudo value 0.5 and then implemented centered log-ratio transformation (Aitchison, 1982) to the relative data. We conducted the same procedure for the real data.

The number of PLS components used for asmbPLS and mbPLS were both taken to be 3 in the simulation study. For asmbPLS, the quantile combinations used for cross validation (CV) in low dimension setting were $quantile^{block_{1}}=quantile^{block_{2}}$ = {0.7, 0.8, 0.9, 0.95, 0.975}; the quantile combinations used for CV in the mixed dimension setting were $quantile^{block_{1}}$ = 0.9, 0.95, 0.975, 0.99, 0.995} and $quantile^{block_{2}}$ = {0.7, 0.8, 0.9, 0.95, 0.975}; and the quantile combinations used for CV in high dimension setting were $quantile^{block_{1}}$ = $quantile^{block_{2}}$ = {0.9, 0.95, 0.975, 0.99, 0.995}. For IPF-Lasso, 9 different penalty ratios were used, i.e. {1:1, 2:1, 1:2, 3:1, 1:3, 4:1, 1:4, 5:1, 1:5}, which indicates different penalty factors, used for two predictor blocks.

##### 3.1.2.1 Prediction performance

The performances of prediction of all the methods were measured in terms of the scaled MS $E_{p}$ using the test set:

$$MSE_{p}=\frac{1}{n_{test}\sigma^{2}}\sum_{i=1}^{n_{test}}{\left({\hat{Y}}_{new,i}-Y_{new,i}\right)}^{2}$$

**FIGURE 2:**
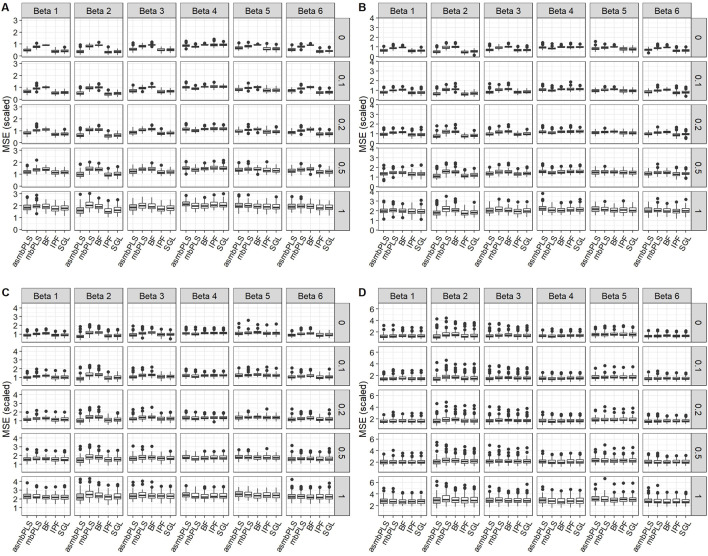
Prediction results for low dimension setting with lognormal distributed survival time and A = 2. **(A)** cr = 0.1; **(B)** cr = 0.3; **(C)** cr = 0.5; **(D)** cr = 0.7. The *x*-axis indicates different methods, and the *y*-axis indicates scaled MSE. The vertical facet titles indicate different β settings, and the horizontal facet titles indicate different noise levels.

Figure 2 displays the results for low dimension setting with lognormal distributed survival time and A = 2. Additionally, we implemented *t*-test to compare the $MSE_{p}$ of asmbPLS with other methods across each scenario, using a significance level of 0.05 as the criterion. The results of Priority-Lasso were not included here due to its much worse performance. For cr = 0.1 (Figure 2A), asmbPLS generally outperforms mbPLS and Block Forest in β settings (1)(2)(3)(5)(6) except in settings (5)(6) with high noise. However, asmbPLS performs worse than the best performer, IPF-Lasso (followed by SGL), in most of the scenarios except in β setting (4) and settings (2)(5) with high noise. In β setting (4), where we assume that all the features have equal contributions to the outcome, mbPLS, considered as a special case of asmbPLS, performs the best. It makes sense since mbPLS makes use of the information from all the features. For cr = 0.3 (Figure 2B), the scaled MSEs of all the methods increase due to the increase in cr . Overall, IPF-Lasso continues to perform the best in most scenarios except in β setting (4). However, the performance of asmbPLS is closer to that of IPF-Lasso in all the scenarios, particularly in high noise conditions. For cr = 0.5 (Figure 2C), the performance of asmbPLS and IPF-Lasso are comparable in most scenarios, and asmbPLS outperform SGL in cases with fewer relevant features such as in β setting (2). For cr = 0.7 (Figure 2D), asmbPLS exhibits slightly better performance than all other methods in β settings (1)(2)(3)(5)(6) with low to moderate noise (r < 1, *p*-value <0.05 compared to mbPLS and Block Forest, and *p*-value >0.05 compared to IPF-Lasso and SGL). In high noise scenarios ( r = 1), all methods show similar performance with no significant differences observed between them. In summary, although asmbPLS is not the best in low censoring rate scenarios, the performance of asmbPLS improves with the increasing censoring rate, and asmbPLS achieves the best performance in high censoring rate scenarios. In addition, asmbPLS performs better in scenarios with fewer relevant features but a higher level of significance of those relevant features, i.e. β setting (2), than the other scenarios. Furthermore, asmbPLS cannot always maintain the best performance with increasing noise.

We observed comparable patterns in low dimension setting with lognormal survival time and A = 0.5 (Additional file 1: Supplementary Figure S1). Moreover, consistent outcomes were noted when the distribution of survival time shifted from lognormal to Weibull (Additional file 1: Supplementary Figures S2, S3). Similarly, in both mixed and high dimension settings, we observed analogous trends with different survival time distribution and values of A (Additional file 1: Supplementary Figures S4–S11).

##### 3.1.2.2 Feature selection

In this section, only results for β settings (1), (2), (3), (5) and (6) are presented since the feature selection for β setting (4) is not necessary. In addition, among all the methods, the feature selection procedure is not included for mbPLS and Block Forest since these methods do not inherently include feature selection. Therefore, we compared asmbPLS with IPF-Lasso, Priority-Lasso, and SGL only. The performances of feature selection were measured in terms of sensitivity and specificity.

For different β settings, we defined the true relevant features differently. For β settings (1), (5) and (6), we defined the true relevant features as the features with positive β . For β settings (2) and (3), since all β s are positive with different values, we defined the true relevant features as the features with false discovery rate (FDR) adjusted *p*-value <0.05 by conducting the univariate linear regression for each feature. After obtaining all the feature selection results, we calculated sensitivity and specificity based on 100 simulated datasets for each scenario.

**FIGURE 3:**
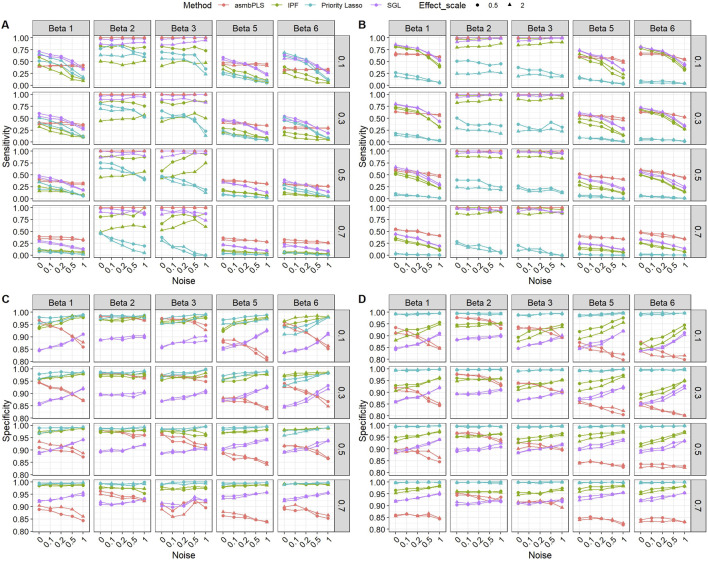
Sensitivity and specificity of the feature selection for low dimension setting with lognormal distributed survival time. **(A)** Sensitivity for microbiome block; **(B)** Sensitivity for metabolome block; **(C)** Specificity for microbiome block; **(D)** Specificity for metabolome block. The *x*-axis indicates different noise levels, and the *y*-axis indicates sensitivity or specificity. The vertical facet titles indicate different β settings, and the horizontal facet titles indicate different censoring rates.

Figure 3 presents the sensitivity and specificity of the feature selection for low dimension setting with lognormal distributed survival time. According to Figure 3, the performances of different methods on feature selection vary in different types of omics data and all methods show a similar trend in different A settings. Regarding the sensitivity (Figures 3A, B), the performance of asmbPLS might not be the best at lower noise with β settings (1), (5) and (6) in scenarios with cr = 0.1, 0.3 or 0.5. However, with the increase of the noise, asmbPLS gradually performs better than the other methods whose sensitivity is largely affected by the noise. For β settings (2) and (3), asmbPLS performs the best with sensitivity close to 1 in all the scenarios regardless of the censoring rate and the noise. This can be due to the definition of the true relevant features in these two β settings because the nature of asmbPLS makes it always find the most significant features (with the lowest *p*-value). For β settings (1), (5) and (6), the true relevant features are defined as the features with positive β . However, it’s important to note that in these scenarios, the feature with positive β may not be significant in the simulations due to the inherent randomness of the simulation process. And this randomness can result in lower sensitivity across all three settings for the methods being evaluated. In scenarios with high censoring rates (0.7), the sensitivity of asmbPLS is the highest among all the methods. This high sensitivity aligns with the lower MSE observed for asmbPLS, highlighting its superiority in scenarios with higher censoring rates. Correspondingly, the better sensitivity of asmbPLS is along with the lower specificity (Figures 3C, D). It is worth noting that the specificity of other methods increases with the increase of noise, there is a gradual decline in the specificity of asmbPLS. This indicates that asmbPLS tends to retain more features compared to other methods, especially in scenarios with higher noise. Retaining at least some features for each block is one of the inherent characteristics of asmbPLS, this nature can enhance the interpretability of results by providing a more comprehensive view of the data. However, it may also have a trade-off with prediction performance in certain cases where including more irrelevant features may introduce noise and potentially hinder the prediction accuracy. For asmbPLS, although with more features included and more false positive counts, the weights are assigned differently for the different blocks and features, which can still help us to find the most significant features. We observed consistent results with Weibull distributed survival time (Additional file 1: Supplementary Figure S12). Similar patterns were also noted across both mixed and high dimension settings (Additional file 1: Supplementary Figures S13–S16).

##### 3.1.2.3 Computational efficiency

Table 4 displays the mean computational time for each method in different dimension settings. The computational time is measured as the time needed for model fitting including the CV. Among all the methods, mbPLS, which does not require the CV step, is the fastest procedure, followed by Priority-Lasso, asmbPLS and IPF-Lasso. As the special case of asmbPLS, the computational time of mbPLS is equal to asmbPLS given selected quantile combinations for all the PLS components. For asmbPLS, the CV step takes the longest time, which largely depends on the number of quantile combinations and the number of folds for the CV. Block Forest and SGL run much slower compared to other methods, especially as the size of data increases. It is noteworthy that IPF-Lasso and Priority-Lasso shows a decreased computational time in the mixed dimension and high dimension settings compared to low dimension setting. The computational time of all the other methods increase in different degrees with the increases of the dimension. SGL and Block Forest are two of the slowest methods among all those evaluated.

**TABLE 4:** Average computation time (in seconds) for different methods in different dimension settings.

Dimension	asmbPLS	mbPLS	Block forest	IPF-lasso	SGL	Priority-lasso
Low	3.33	0.15	182.18	5.18	105.80	0.70
Mixed	5.54	0.27	280.29	4.10	297.61	0.63
High	7.29	0.37	359.62	4.85	557.19	0.60

### 3.2 Simulation study 2

#### 3.2.1 Simulation strategies

We utilized a simulated multi-omics data generated with OmicsSIMLA (Chung and Kang, 2019), based on the ovarian cancer data from the TCGA project (https://omicssimla.sourceforge.io/simuomicsTCGA.html). OmicsSIMLA, a multi-omics data simulator, can simulate various types of omics data while modeling the relationships between omics data. 50 batches of samples were simulated, with each batch containing normalized RNA-seq data of 12,000 genes and normalized proteomics data of 12,000 proteins across 1,000 patients, comprising 500 cases and 500 controls. For simplicity, we selected 500 cases and the first 2,000 features from each omics data in each batch. In each batch, we utilized RNA-seq data and proteomics data to generate the censored survival time as described in section “Censored Survival Time”, with the first 400 patients treated as the training set and the remaining 100 patients as the test set. Among the 400 patients in the training set, 50% of the patients were designated to be censored. In each type of omics data, only the first 5 features were set as relevant, with the coefficients of all remaining features set to zero. The noise to signal ratio, r , was set to 0.5. SGL was excluded from the comparison because of its extensive computation time.

#### 3.2.2 Simulation results

The numbers of PLS components used for asmbPLS and mbPLS were both taken to be 3 in the simulation study. For asmbPLS, the quantile combinations used for cross validation (CV) were $quantile^{block_{1}}=quantile^{block_{2}}$ = {0.95, 0.96, 0.97, 0.98, 0.99, 0.995}. For IPF-Lasso, 9 different penalty ratios were used, i.e. {1:1, 2:1, 1:2, 3:1, 1:3, 4:1, 1:4, 5:1, 1:5}, which indicates different penalty factors used for two predictor blocks.

Table 5 outlines the comparative results for prediction and feature selection capabilities. Regarding prediction, IPF-Lasso is the top performer among the five evaluated methods, closely followed by asmbPLS (no significant difference between IPF-Lasso and asmbPLS). Conversely, mbPLS and Block Forest are noted for their inferior performance. For feature selection, asmbPLS and IPF-Lasso each achieve perfect sensitivity for RNA-seq data, with Priority-Lasso showing a sensitivity of 0.84, albeit with a higher specificity. For proteomics data, Priority-Lasso has the highest sensitivity, followed by asmbPLS, while IPF-Lasso’s sensitivity is markedly lower. Notably, Priority-Lasso attains the highest specificity for proteomics data also. Overall, asmbPLS demonstrates the highest average sensitivity across both omics datasets, with its prediction performance close to the best.

**TABLE 5:** Simulation results comparison regarding prediction and feature selection for simulation study 2. The results are the average of the 50 batches.

Method	$MSE_{p}$	RNA-seq		Proteomics	
		Sensitivity	Specificity	Sensitivity	Specificity
asmbPLS	1.10	1	0.988	0.112	0.995
mbPLS	1.68	-	-	-	-
Block Forest	1.68	-	-	-	-
IPF-Lasso	1.08	1	0.986	0.044	0.998
Priority-Lasso	1.26	0.84	0.999	0.164	1

### 3.3 Application to melanoma patients data

In our study, we utilized data from Aitchison (1982), encompassing progression-free survival (PFS) interval/status, clinical variables, and diverse omics data gathered from 167 melanoma patients. We focused on three blocks of data: one low-dimensional block comprising clinical covariates, and two high-dimensional blocks consisting of microbiome data and proteomics data. The outcome of interest is the progression-free survival interval, with patients experiencing unobserved events (those without disease progression or death detection) considered right-censored. Among the available clinical variables, we selected 17 clinical variables, age and BMI are continuous with all the other variables are binary: sex, tumor response status, types of treatment, substage of disease in patients with late-stage disease, dietary fiber intake level, lactate dehydrogenase (LDH) level, probiotics use at baseline, antibiotic use at baseline, metformin use at baseline, steroid use at baseline, statin use at baseline, proton pump inhibitor (PPI) use at baseline, beta-blocker use at baseline, other (non-beta-blocker) hypertensive medication use at baseline, whether or not the patient received system therapy prior to baseline. The microbiome block and the proteomics block contain 225 microbial features and 6,773 proteins, respectively.

After filtering the subjects with missing values and without survival data, we obtained 89 samples (55 events) for the downstream implementation. The mean imputation was conducted first for the censored survival time, we then applied the AFT model to determine whether there is an association between PFS and any of the features in the three blocks. Since we have no prior information about the association, we implemented a univariate analysis for each feature to determine whether any of the features are predictive of PFS. To this end, we fitted the simple linear regression model with imputed log-survival PFS as our outcome and each feature as our predictor one at a time. The obtained *p*-values in each block were then adjusted using the false discovery rate (FDR). Specifically, at a significance level of 5%, no microbial features and proteins are significant. For clinical variables, only tumor response status and substage of disease are significant. With this information, we applied asmbPLS and the other methods in the real data and compared them in three aspects: 1) The fit of the model to data; 2) Prediction error of the model; 3) Feature selection of the model.

To measure the fit of the model to data, we used the MSE of fit to compare different methods:

$$\text{MS}E_{F}=\frac{1}{n_{O}}\sum_{i=1}^{n}\delta_{i}{\left({\hat{Y}}_{i}-Y_{i}\right)}^{2}$$

where $n_{O}$ is the number of observed samples and $\delta_{i}$ is the event indicator for sample i . We included the first three PLS components for comparison for PLS-based methods. In addition, for asmbPLS, the 5-fold CV was implemented to obtain the best quantile combination used for model fitting. The pre-determined quantile combinations set were $quantile^{microbiome}$ = {0.9, 0.925, 0.95, 0.975, 0.99, 0.999}, $quantile^{proteomics}$ = {0.997, 0.9985, 0.9993, 0.9999}, $quantile^{clinical}$ = {0, 0.3, 0.5, 0.7, 0.8, 0.9, 0.99}, resulting in 168 combinations considered. Based on the results of CV, the optimal number of PLS components is 1, combination (0.999, 0.9999, 0.9) was selected for the first PLS component. Table 6 lists the values of $MSE_{F}$ . Among all the methods, asmbPLS shows the lowest $MSE_{F}$ , followed by SGL, IPF-Lasso, mbPLS, and Block Forest. The performance of Priority-Lasso is much worse than all the other methods, which is corresponding to the results from the simulation study. On the other hand, to evaluate the prediction performance of the model, we computed the MSE of prediction via leave-one-out (LOO) CV with each observed sample serving as validation data for once:

$$\text{MS}E_{P,LOO}=\frac{1}{n_{O}}{\sum_{i=1}^{n}\delta_{i}\left({\hat{Y}}_{i,-}-Y_{i}\right)}^{2}$$

where ${\hat{Y}}_{i,-}$ is computed by the model using the dataset with i th sample excluded. The selected quantile combination from the previous CV was used here for the validation set. The results are listed in Table 6. Block Forest was able to fit the model, but cannot be used to do the prediction due to the error message in R. As seen in the table, among all the other methods, SGL performs the best, followed by IPF-Lasso, asmbPLS, mbPLS, and Priority-Lasso.

**TABLE 6:** Comparison of the model fitting and prediction performance for different methods using the melanoma patient data.

Methods	$MSE_{F}$	$MSE_{P,LOO}$
asmbPLS	0.609	0.817
mbPLS	0.736	1.451
Block Forest	1.096	-
IPF-Lasso	0.658	0.792
SGL	0.644	0.782
Priority-Lasso	19.949	21.766

For asmbPLS, 1 microbial taxon, 1 protein, and 2 clinical variables were selected. And block weights for microbiome block, proteomics block, and clinical blocks were 0.069, 0.021, and 0.997, respectively. Due to the nature of asmbPLS, at least one feature was selected for each block. Nevertheless, the block weights for microbiome and proteomics data were notably less than that of the clinical block. This suggests that clinical features could be more relevant to the outcome, which was validated by the univariate analysis. The two clinical variables selected by asmbPLS were tumor response status and substage of disease, which were the only two significant clinical variables. Furthermore, all features identified by asmbPLS within the microbiome and proteomics blocks were significant prior to FDR adjustment. Notably, within the microbiome block, *Ruminococcus lactaris* stands out. It is worth highlighting that elevated levels of *R. lactaris* are associated with extended PFS and exhibit a tendency towards reduced systemic inflammation, as evidenced in a B cell-lymphoma patient group (Stein-Thoeringer et al., 2023). Our study corroborates these findings, indicating that increased levels of *R. lactaris* correspond to prolonged PFS among melanoma patients. Moreover, in the proteomics block, the selected feature is NADH oxidase, which plays a pivotal role in regulating growth and transcription in melanoma cells (SS et al., 2002).

For IPF-Lasso, 0 microbial feature, 0 protein, and 2 clinical variables were selected. The variable substage of disease, which was highly significant before and after FDR adjustment, was not selected by IPF-Lasso. Conversely, the lactate dehydrogenase (LDH) level, which was not significant prior to FDR adjustment, was selected. For SGL, 0 microbial feature, 0 protein, and 3 clinical variables were selected. In addition to the two significant variables, LDH level was also selected by SGL and assigned with a much higher weight than substage of disease. For priority-Lasso, only tumor response status was selected.

**FIGURE 4:**
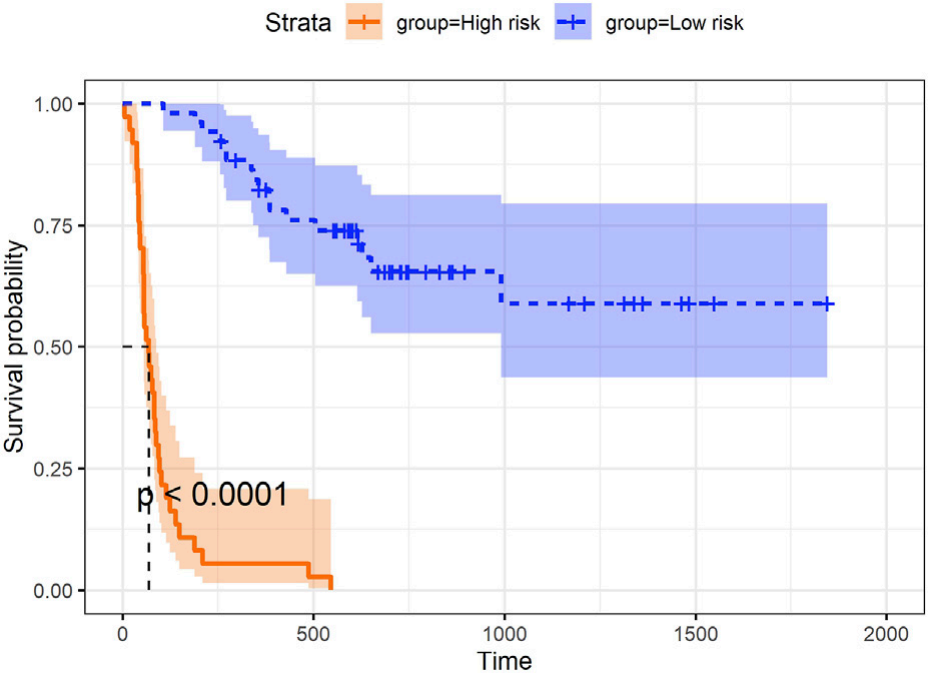
Prediction of PFS using super score from asmbPLS.

In addition to the three aspects discussed above, we explored the utility of the super score, which integrated information from all data blocks (e.g., microbiome, proteomics, and clinical data) and was uniquely derived using asmbPLS and mbPLS, as the predictor for the outcome. While asmbPLS selected a limited number of features, it prioritized those with the strongest and most relevant contributions to the outcome, enabling the super score to capture the most critical information from each block. Specifically, an optimal cut-point on super score was determined to define the two groups using the maximally selected rank statistics (Lausen and Schumacher, 1992) as implemented in the R package *survminer* (Kassambara et al., 2017), and the *p*-value was calculated based on a log-rank test between the resulting groups. As seen in Figure 4, super score is significantly associated with progression-free survival time. Patients with higher super scores (blue group) seem to have much higher survival probability. In other words, once we have a new sample with its corresponding microbiome, proteomics, and clinical data, asmbPLS can calculate the super score and then assign the sample to the high or low risk group. This information could be instrumental in designing personalized treatment tailored to each patient.

Furthermore, we have done the additional analysis without using the most significant clinical block. In this test, only microbiome block and proteomics are included as predictors. The same pre-determined quantile combinations set are used for microbiome and proteomics blocks. Based on the results of CV, the optimal number of PLS components is 1, combination (0.999, 0.9999) was selected for the first PLS component, indicating that 1 microbial taxon and 1 protein were selected. These two features are still the most significant feature in each block even though they are not significant after *p*-value adjustment. For the other three Lasso-based methods, 0 microbial taxon and 0 protein was selected. Table 7 lists the results for comparison of model fit and CV. With the fact that no predictor is relevant, all the methods show relatively higher $MSE_{P,LOO}$ than in Table 6. Especially, asmbPLS shows higher $MSE_{P,LOO}$ than the other Lasso-based methods, which is due to the nature of asmbPLS, which always keeps at least one most relevant predictor for each block. This nature will help the prediction when there are significant predictors but can make the prediction worse if there is no real relevant predictor.

**TABLE 7:** Comparison of the model fitting and prediction performance for different methods using the melanoma patient data (without clinical block).

Methods	$MSE_{F}$	$MSE_{P,LOO}$
asmbPLS	2.21	2.94
mbPLS	1.31	3.45
Block Forest	2.49	-
IPF-Lasso	2.35	2.26
SGL	2.35	2.24
Priority-Lasso	2.35	2.24

### 3.4 Application to TCGA-LUSC data

In addition, we applied TCGA-LUSC data to further assess the feature selection capability of asmbPLS. Lung squamous cell carcinoma is a type of non-small cell lung cancer (NSCLC). Among NSCLC, adenocarcinoma is the most common, followed by squamous cell carcinoma of the lung, especially in women (Sabbula et al., 2023). The analysis included gene expression, miRNA and survival data. Subjects with missing gene, miRNA or survival data were excluded, resulting in a final dataset of 362 subjects (91 events) with 45,362 genes and 1,409 miRNAs. Similarly, mean imputation was performed for the censored survival time, and asmbPLS was then applied on the dataset. For comparison, IPF-Lasso was also conducted. SGL and Priority-Lasso were excluded from the analysis as they generated error messages during the model fitting process. We included the first three PLS components for asmbPLS and 5-fold CV was used to determine the optimal quantile combination for model fitting. The pre-determined quantile combinations set were $quantile^{gene}$ = {0.9997, 0.9998, 0.9999, 0.99999}, $quantile^{miRNA}$ = {0.99, 0.995, 0.999}. Based on the results of CV, the optimal number of PLS components is 1, combination (0.99999, 0.999) was selected for the first PLS component.

Regarding feature selection, 1 gene (ENSG00000286152) and 2 miRNAs (has-mir-610 and has-mir-7977) were selected by asmbPLS, with block weights of 0.194 for the gene block and 0.981 for the miRNA block, indicating that the selected miRNAs might be more relevant. For the selected gene, ENSG00000286152, does not currently have an associated gene symbol in available databases. However, for selected miRNAs, miR-610 was found to suppress lung cancer cell proliferation and invasion by targeting GJA3 expression (Jin et al., 2014), while exosomal miR-7977 has been identified as a novel biomarker for patients with lung adenocarcinoma and may function as a tumor suppressor in lung cancer (Chen et al., 2020). No feature was selected by IPF-Lasso.

## 4 Discussion and conclusion

In this paper, we developed asmbPLS algorithm to identify the most significant features of the multi-omics data gathered from the same set of samples. Subsequently, these selected features are harnessed for outcome prediction. Different from conventional smbPLS, asmbPLS is flexible in determining the penalty factor for different omics data in different PLS components. With some prior knowledge of omics data, the pre-decided quantile set can be provided to each block. Then, the best quantile combination can be chosen in a completely data-driven manner. In addition, using the quantile makes the interpretation more straightforward, block with selected quantile = 0.95 indicates that only the top 5% features are relevant to the outcome. asmbPLS works with continuous predictor variables and continuous outcomes, binary variables can be transformed to 0/1 to meet the requirement. And for categorical variables with more than 2 levels, the one-hot encoding can be one strategy, where the categorical variable with *G* levels can be transformed into *G* - 1 dummy variables. asmbPLS is implemented in the R package *asmbPLS* available on our GitHub (https://github.com/RunzhiZ/asmbPLS).

Simulation study 1 has demonstrated the superior predictive performance of asmbPLS compared to other methods, particularly in scenarios characterized by higher censoring rates, especially when dealing with fewer relevant features such as β setting (2). However, in scenarios with low to moderate censoring rate (0.1, 0.3 and 0.5), asmbPLS does not outperform IPF-Lasso. Additionally, as noise increases, the MSE of asmbPLS rises similarly to other methods, and the difference in MSE between methods become smaller. In scenarios with more relevant features, such as β settings (4)(5)(6), asmbPLS tends to have higher MSE. This occur because including more related features in the survival time simulation reduces the individual effect of each feature, as β is normalized to control the overall effect, making it more difficult to identify the relevant features. According to the results of the simulation study, including three PLS components may suffice for balanced prediction performance and computational efficiency, since, in most simulations, the optimal number of PLS components is either 1 or 2. Including more PLS components fails to consistently improve prediction accuracy while incurring additional computational demands in the CV procedure. Regarding feature selection, asmbPLS outperforms other methods in identifying truly relevant features, especially in scenarios with high noise and censoring rates. This aligns with the essence of asmbPLS, where significant features are consistently identified. This corresponds to the results from β settings (2) and (3), where truly relevant features are defined as those with an adjusted *p*-value <0.05 using univariate linear regression. Although asmbPLS tends to select a relatively larger number of features compared to other methods, the allocated weights remain informative for both the features and the blocks, proving effective in identifying the most significant contributors. In simulation study 2, asmbPLS continues to exhibit nearly the best prediction performance while delivering the top performance in feature selection.

The performance of asmbPLS is further validated through its application to melanoma patient data. Notably, asmbPLS retained features from both the microbiome and proteomics blocks, even in cases where no significant feature was identified (after *p*-value adjustment) within these blocks. The calculated block weights of 0.069, 0.021, and 0.997 validate the limited contributions of the microbiome and proteomics blocks. Furthermore, it is noteworthy that despite the retention of features, the top features selected by asmbPLS from the microbiome and proteomics blocks exhibit meaningful biological significance. In addition, the exploration of the super score in melanoma patient data has demonstrated that super score of asmbPLS is an effective predictor for classifying different survival groups. This practice holds significant potential, enabling the assignment of patients into high-risk or low-risk groups, thereby facilitating the development of personalized treatment plans. In addition to the melanoma dataset, we applied asmbPLS to the TCGA-LUSC dataset, which includes gene expression, miRNA, and survival data, to further evaluate its feature selection capability in lung cancer. asmbPLS successfully identified 1 gene and 2 miRNAs as key features, with miRNAs appearing to have higher relevance based on block weights. This further underscores asmbPLS’s ability to select biologically meaningful features, outperforming other methods like IPF-Lasso, which failed to select any features in this dataset.

A notable limitation of asmbPLS emerges when there is no real relevant feature in the predictor blocks. In such cases, the inclusion of a certain number of features within each block might sacrifice the predictive performance of asmbPLS, although enhancing our understanding of the relative importance of these features. In addition, to perform the model fitting in asmbPLS, each subject must have the same number of predictors and outcomes with no missing data. If any predictors contain missing values, the affected samples must be removed before model fitting, which can reduce the effective sample size and potentially bias the results. This limitation may be particularly problematic in datasets with a high proportion of missing data, as it can lead to a loss of statistical power and hinder the model’s ability to generalize. Future enhancements could include integrating imputation methods to better manage missing data. Furthermore, while mean imputation offers computational convenience for handling right censored survival data, it has the potential to introduce bias, especially in cases where the censoring mechanism is not random. In future extensions of this work, it’s worth to explore the impact of different imputation methods, such as reweighting and multiple imputation (Datta et al., 2007), on the prediction performance of asmbPLS.

In summary, by integrating multi-omics data and continuous phenotypes, asmbPLS can identify the most relevant features across various omics layers and utilizes these selected features for prediction. asmbPLS delivers competitive performance in prediction, feature selection, and computational efficiency compared with other state-of-the-art methods. We anticipate asmbPLS to be a valuable tool for multi-omics research.

## Data Availability

The datasets presented in this study can be found in online repositories. The names of the repository/repositories and accession number(s) can be found below: https://github.com/RunzhiZ/Runzhi_Susmita_asmbPLS_2024.
