# Supplementary material for: asmbPLS: biomarker identification and patient survival prediction with multi-omics data
Source: Front Genet. 2024 Nov 22;15:1444054. doi: 10.3389/fgene.2024.1444054 (PMC11621212; doi:10.3389/fgene.2024.1444054)
Supplement: Supplementary file 1 [file Table1.docx]

Supplementary Material for “asmbPLS: Biomarker Identification and Patient Survival Prediction with Multi-omics Data”

Runzhi Zhang^1^, Susmita Datta^1*^

^1^Department of Biostatistics, University of Florida, Gainesville, FL, United States

*** Correspondence:** [susmita.datta@ufl.edu](mailto:susmita.datta@ufl.edu)

**Supplementary Figures**

**
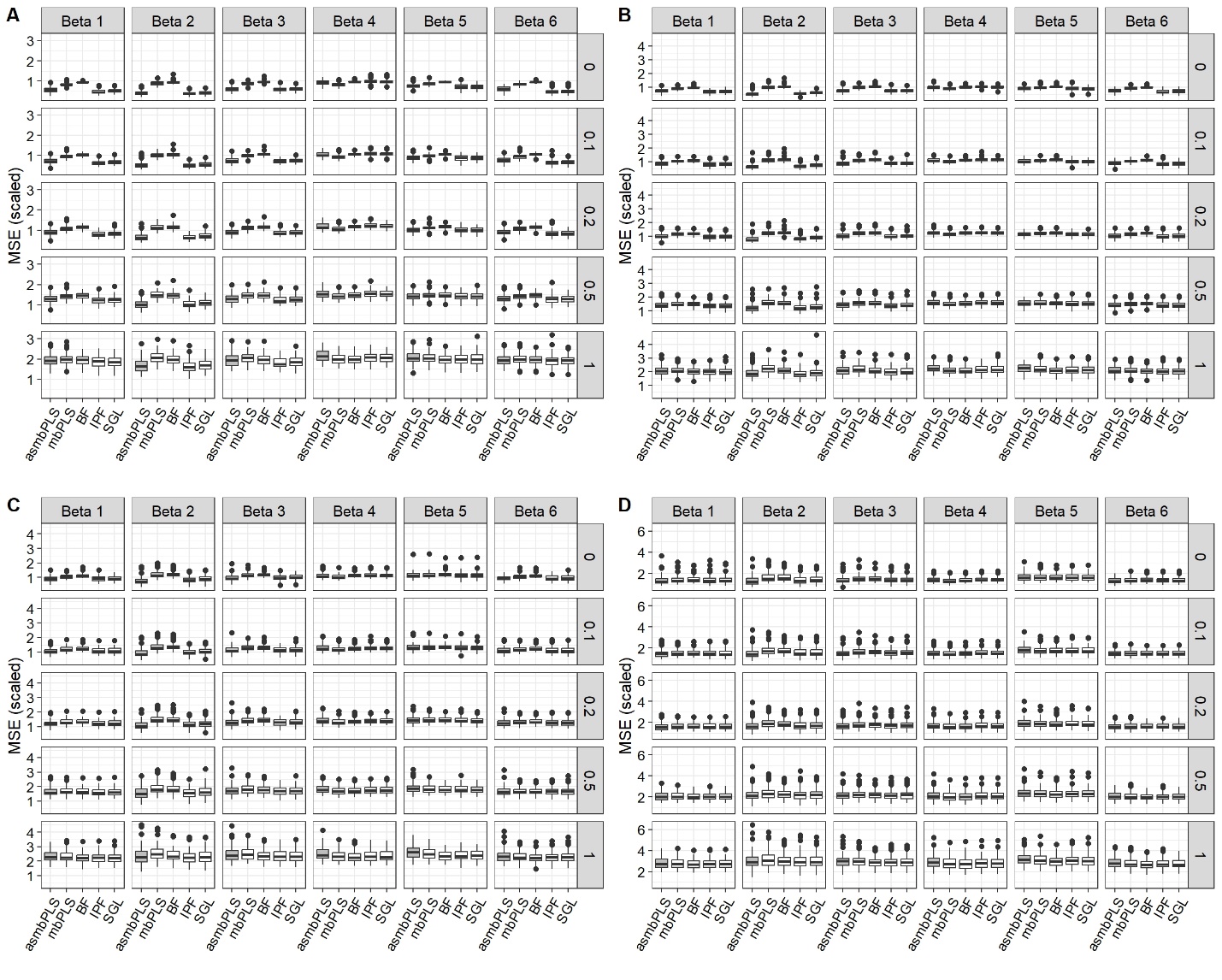
**

**Figure S1**. Prediction results for low dimension setting with lognormal distributed survival time and $A$ = 0.5. **(A)** $cr$ = 0.1; **(B)** $cr$ = 0.3; **(C)** $cr$ = 0.5; **(D)** $cr$ = 0.7.

**
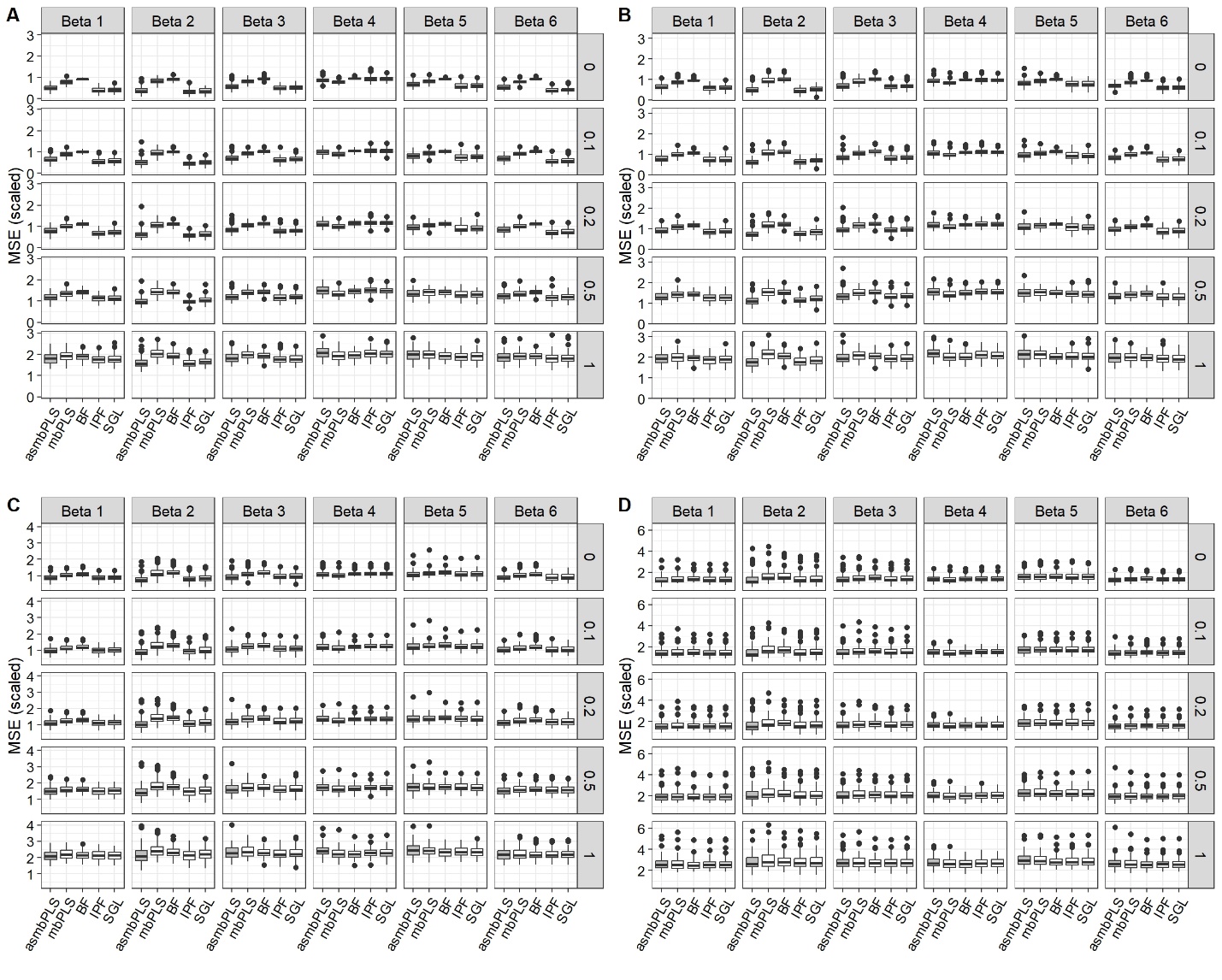
**

**Figure S2**. Prediction results for low dimension setting with Weibull distributed survival time and $A$ = 2. **(A)** $cr$ = 0.1; **(B)** $cr$ = 0.3; **(C)** $cr$ = 0.5; **(D)** $cr$ = 0.7.

**
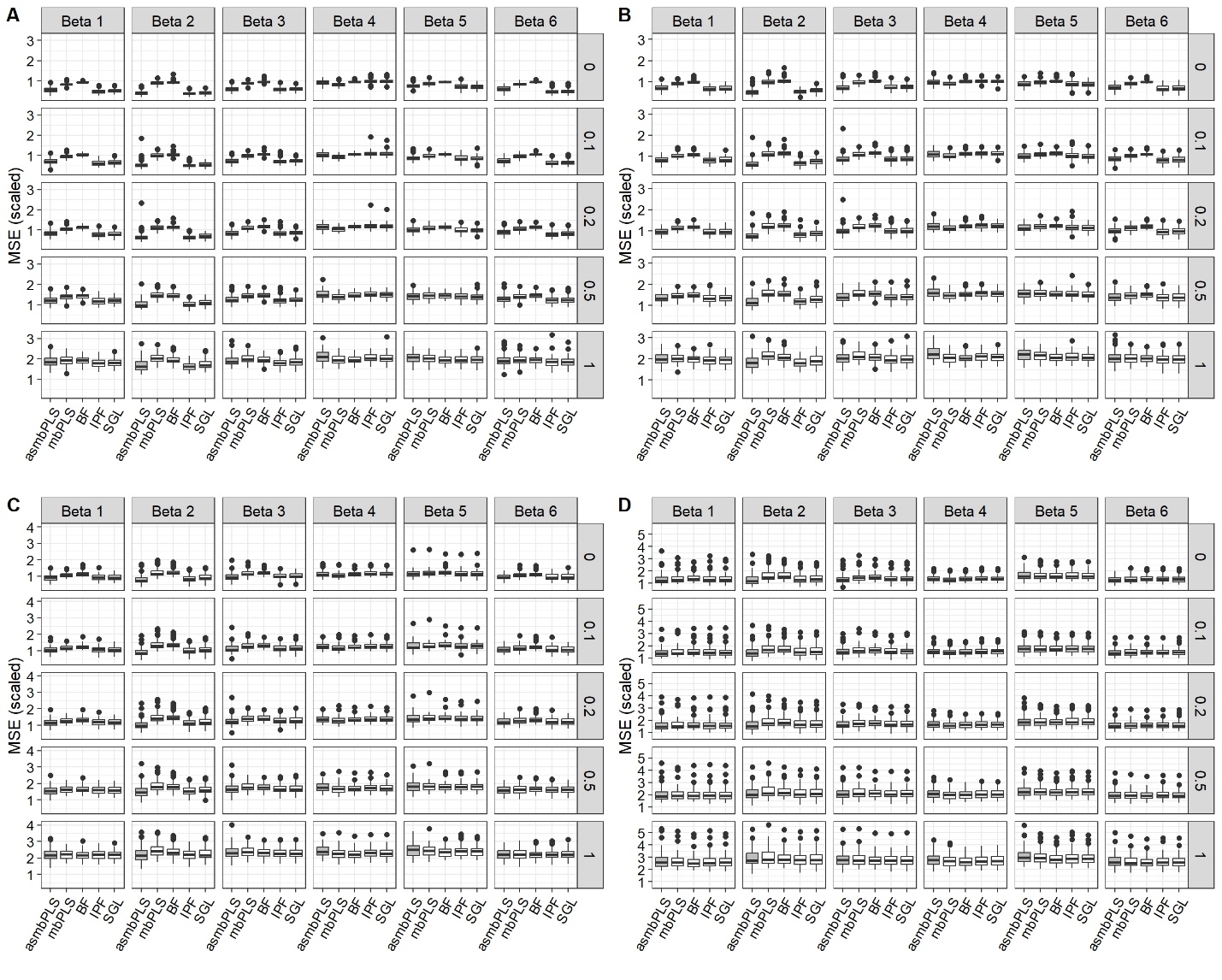
**

**Figure S3**. Prediction results for low dimension setting with Weibull distributed survival time and $A$ = 0.5. **(A)** $cr$ = 0.1; **(B)** $cr$ = 0.3; **(C)** $cr$ = 0.5; **(D)** $cr$ = 0.7.

**
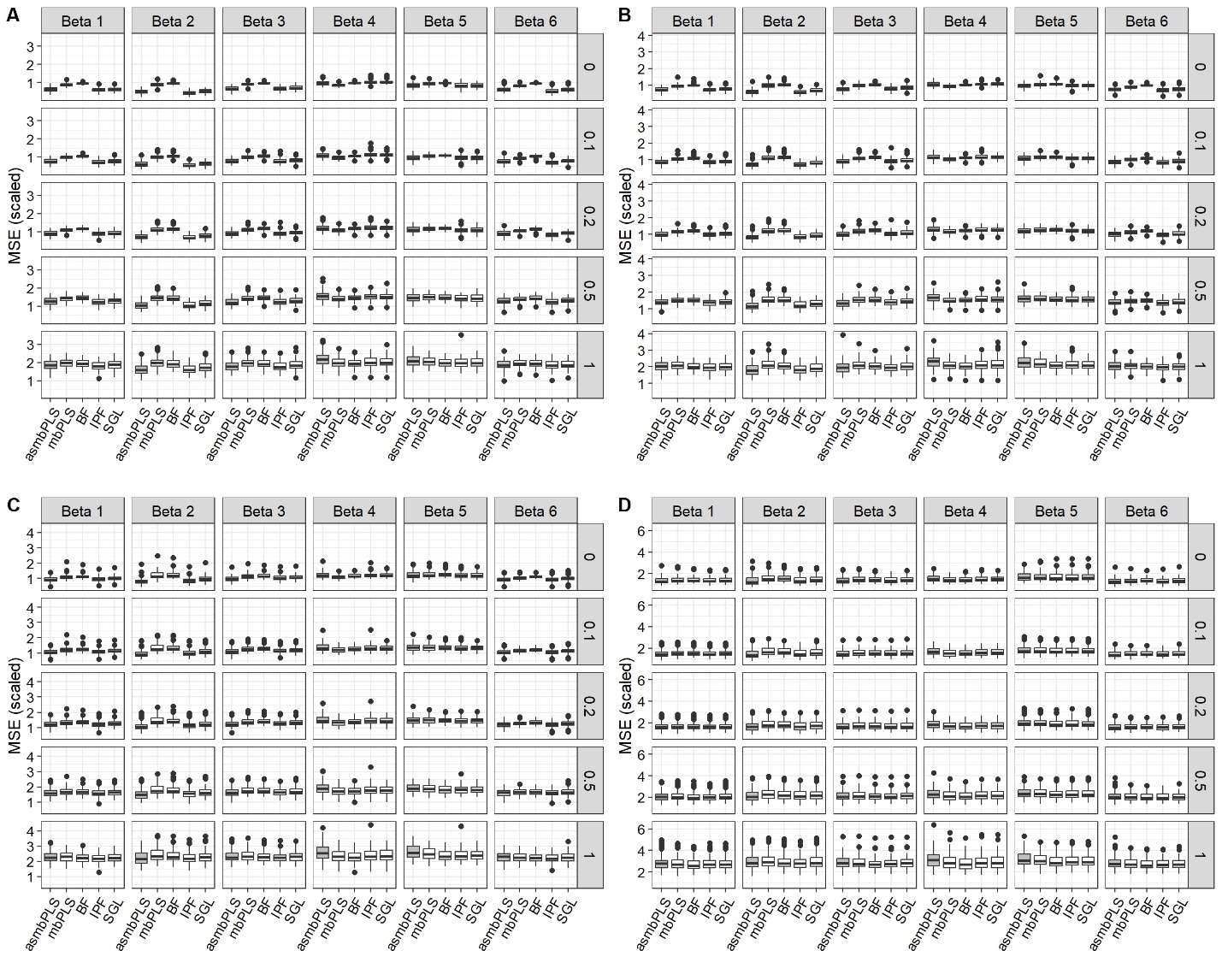
**

**Figure S4**. Prediction results for mixed dimension setting with lognormal distributed survival time and $A$ = 2. **(A)** $cr$ = 0.1; **(B)** $cr$ = 0.3; **(C)** $cr$ = 0.5; **(D)** $cr$ = 0.7.

**
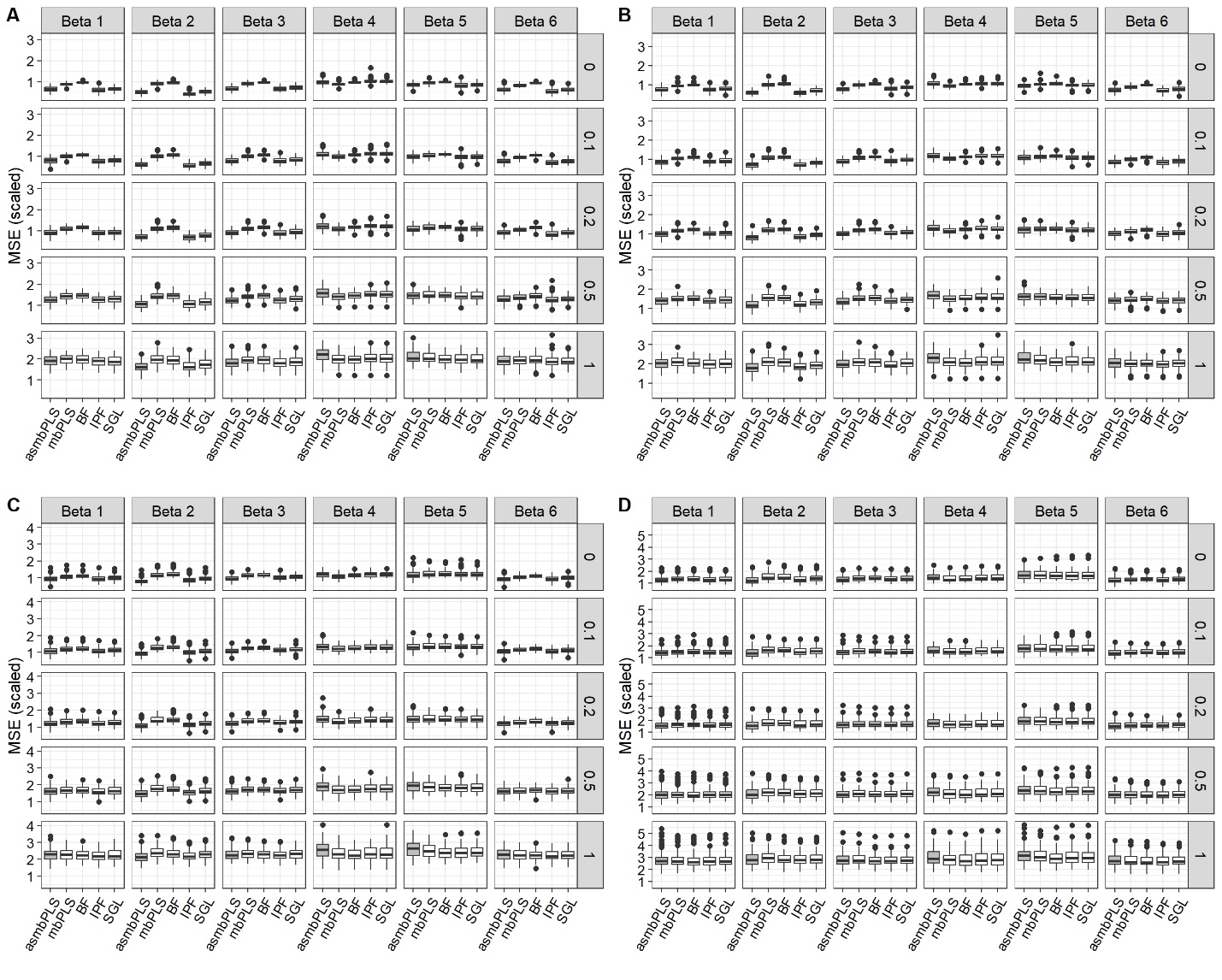
**

**Figure S5**. Prediction results for mixed dimension setting with lognormal distributed survival time and $A$ = 0.5. **(A)** $cr$ = 0.1; **(B)** $cr$ = 0.3; **(C)** $cr$ = 0.5; **(D)** $cr$ = 0.7.

**
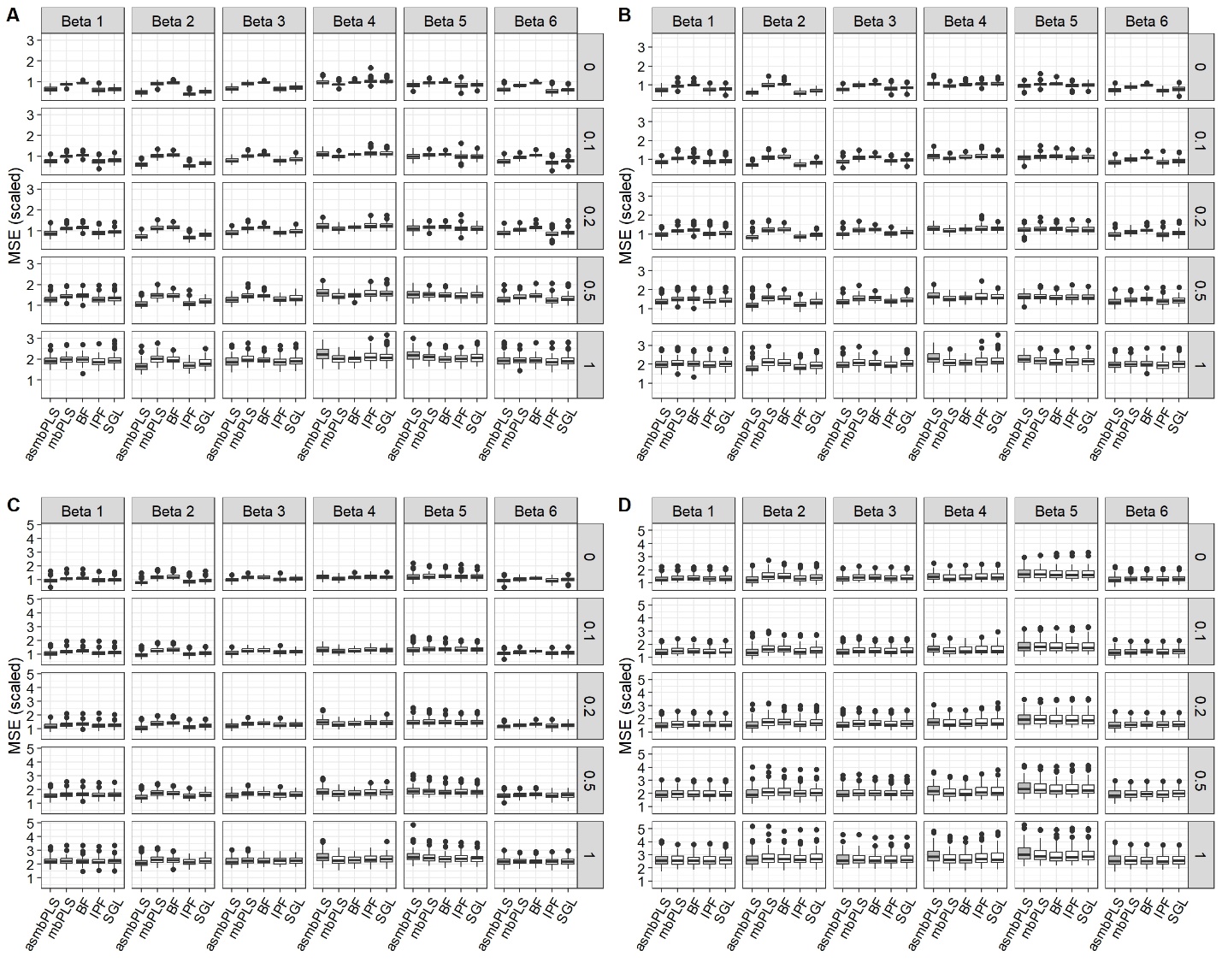
**

**Figure S6**. Prediction results for mixed dimension setting with Weibull distributed survival time and $A$ = 2. **(A)** $cr$ = 0.1; **(B)** $cr$ = 0.3; **(C)** $cr$ = 0.5; **(D)** $cr$ = 0.7.

**
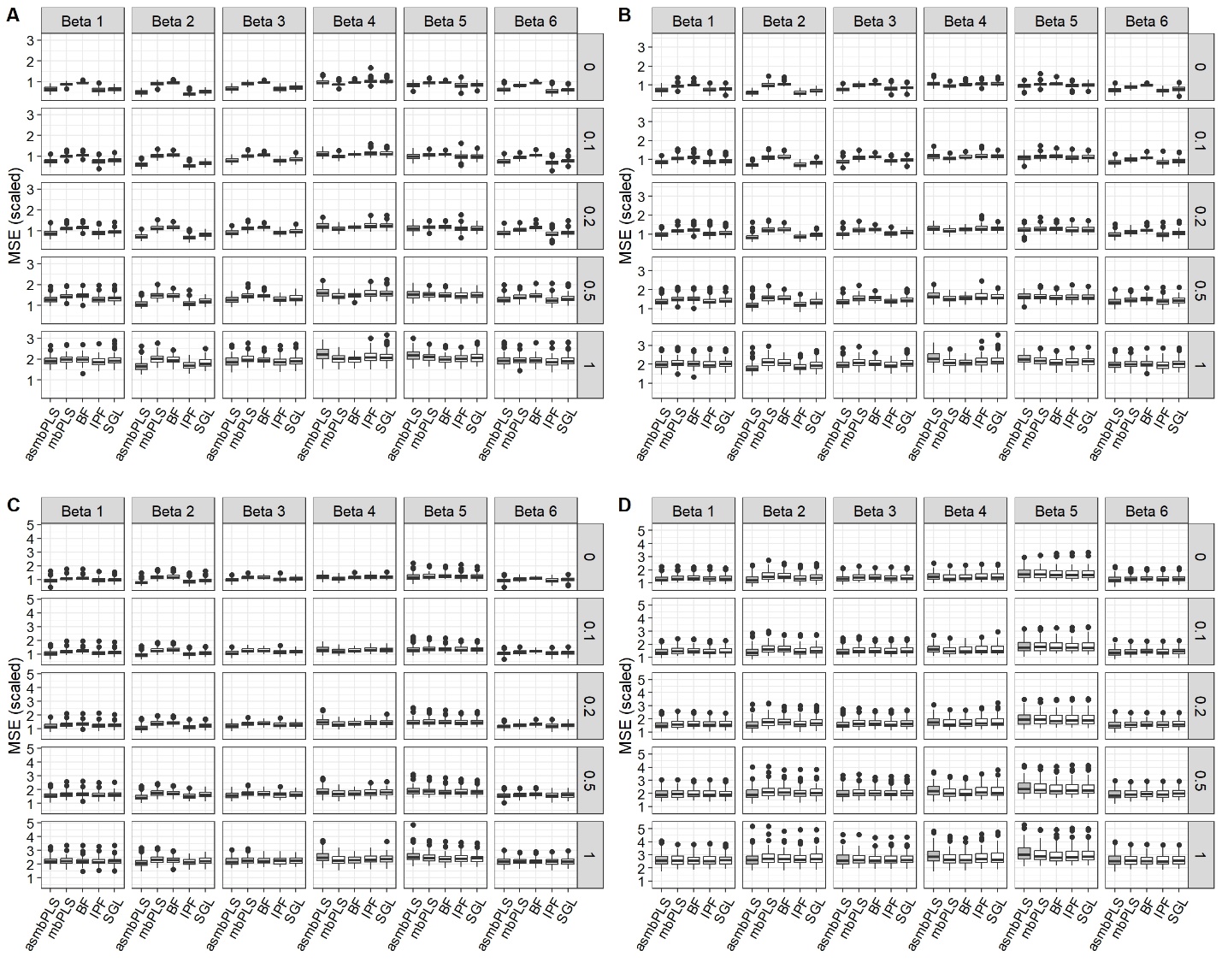
**

**Figure S7**. Prediction results for mixed dimension setting with Weibull distributed survival time and $A$ = 0.5. **(A)** $cr$ = 0.1; **(B)** $cr$ = 0.3; **(C)** $cr$ = 0.5; **(D)** $cr$ = 0.7.

**
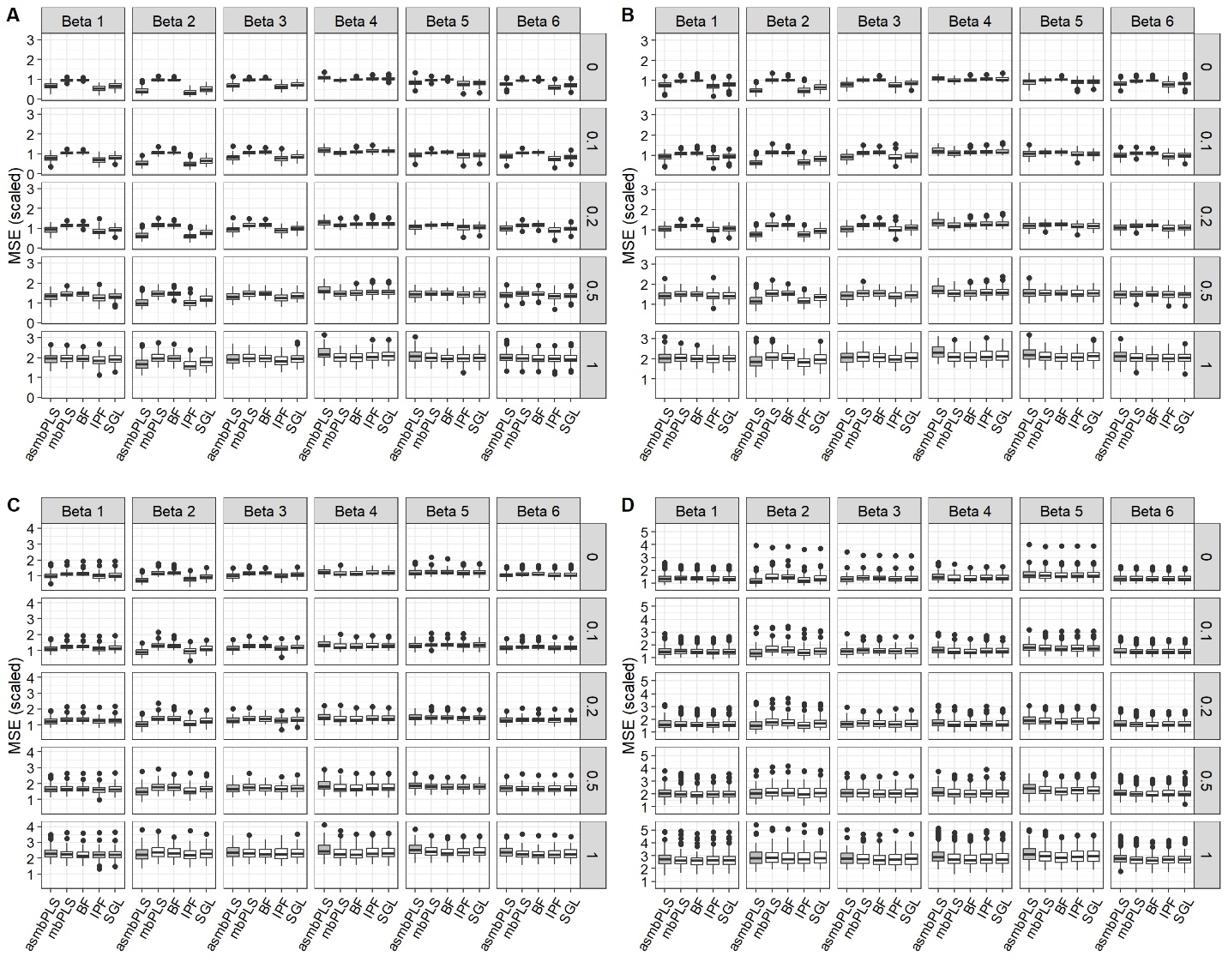
**

**Figure S8**. Prediction results for high dimension setting with lognormal distributed survival time and $A$ = 2. **(A)** $cr$ = 0.1; **(B)** $cr$ = 0.3; **(C)** $cr$ = 0.5; **(D)** $cr$ = 0.7.

**
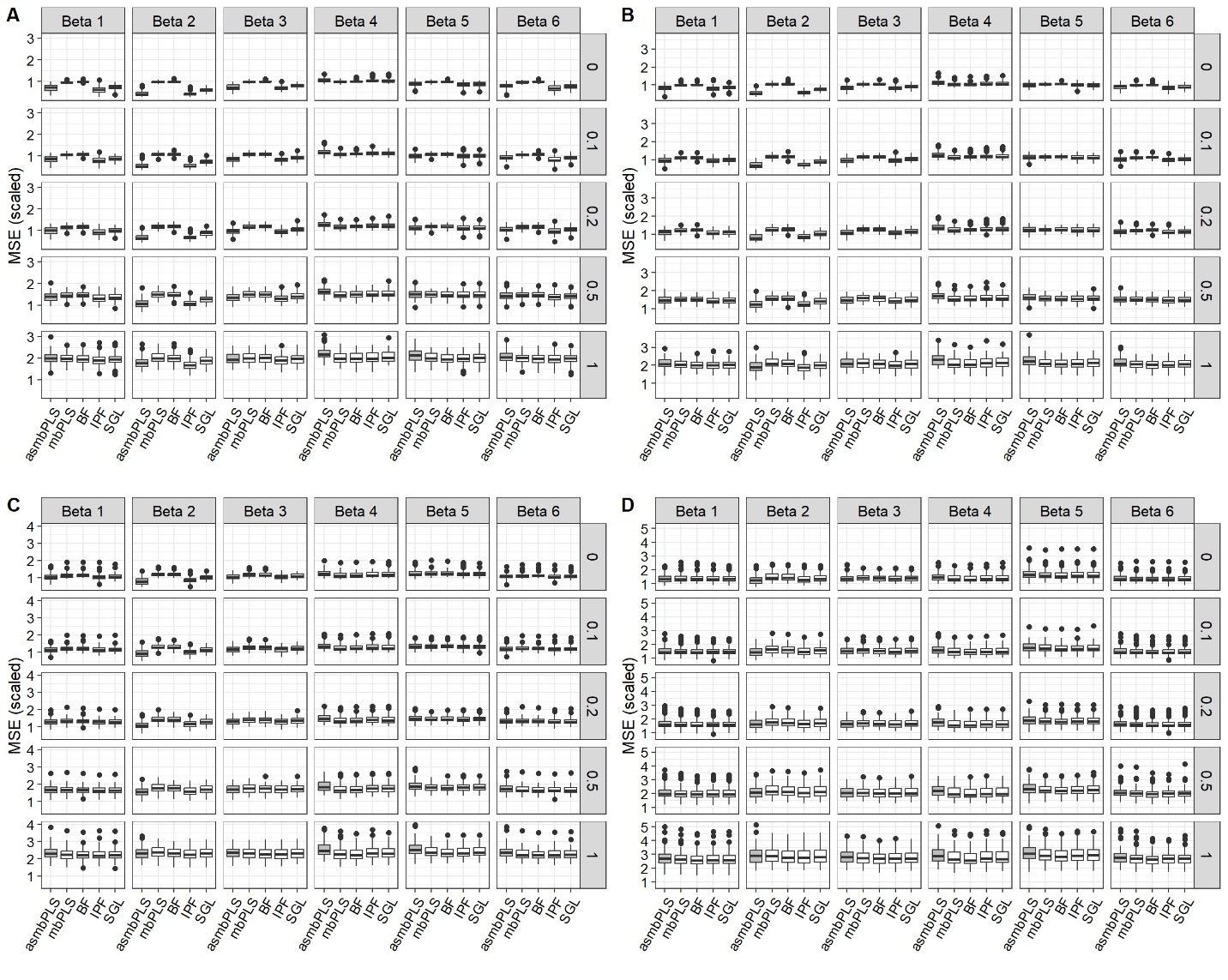
**

**Figure S9**. Prediction results for high dimension setting with lognormal distributed survival time and $A$ = 0.5. **(A)** $cr$ = 0.1; **(B)** $cr$ = 0.3; **(C)** $cr$ = 0.5; **(D)** $cr$ = 0.7.

**
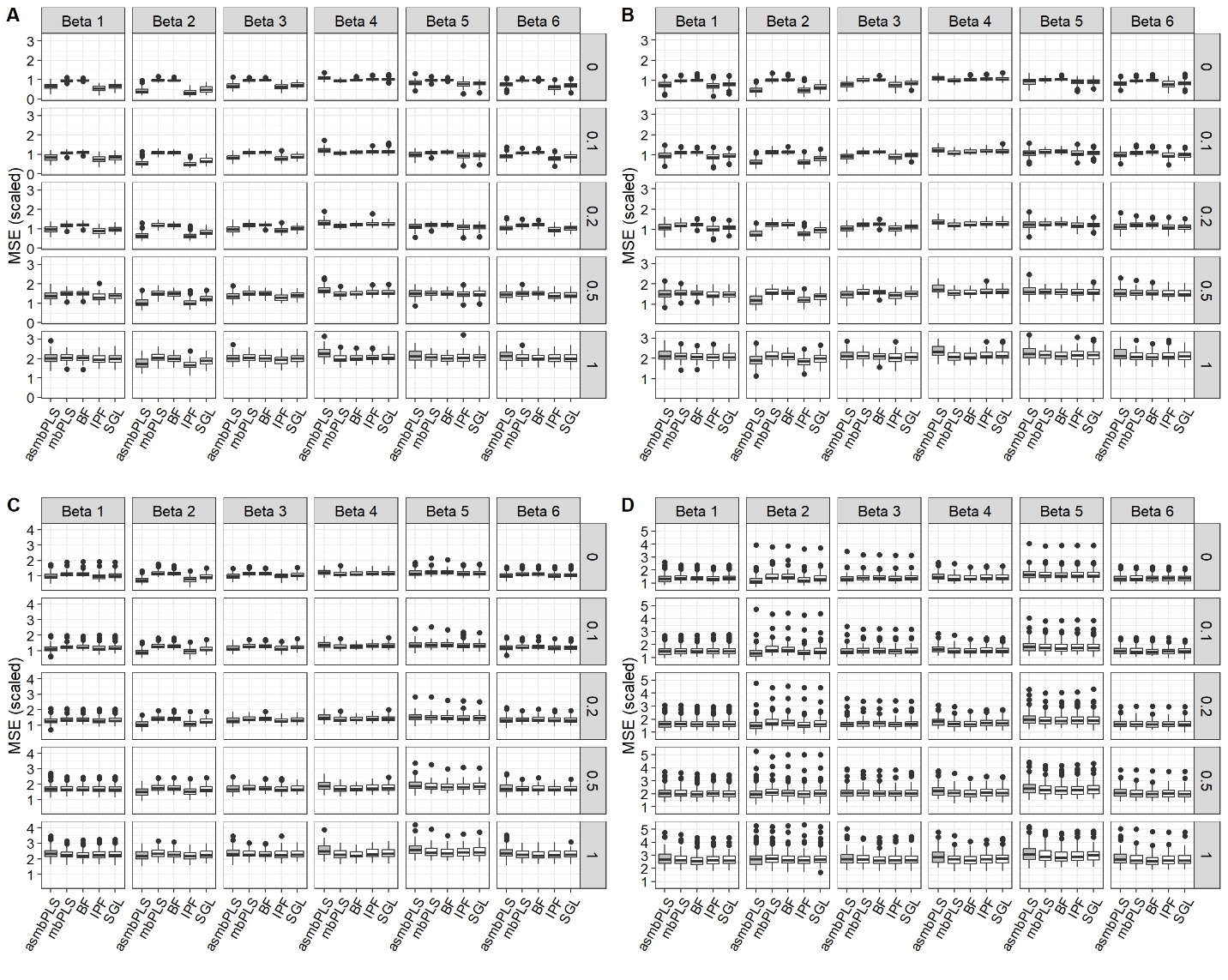
**

**Figure S10**. Prediction results for high dimension setting with Weibull distributed survival time and $A$ = 2. **(A)** $cr$ = 0.1; **(B)** $cr$ = 0.3; **(C)** $cr$ = 0.5; **(D)** $cr$ = 0.7.

**
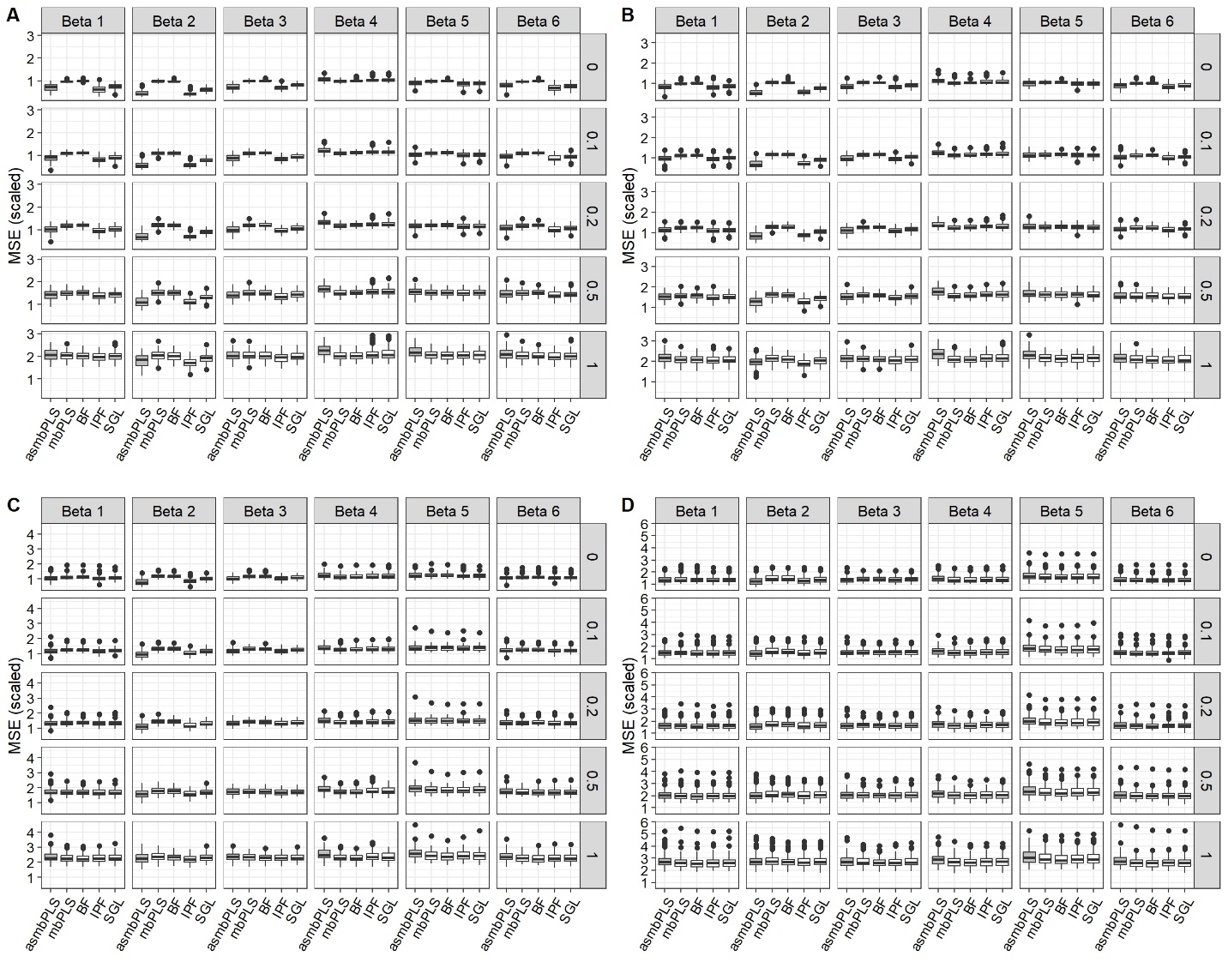
**

**Figure S11**. Prediction results for high dimension setting with Weibull distributed survival time and $A$ = 0.5. **(A)** $cr$ = 0.1; **(B)** $cr$ = 0.3; **(C)** $cr$ = 0.5; **(D)** $cr$ = 0.7.

**
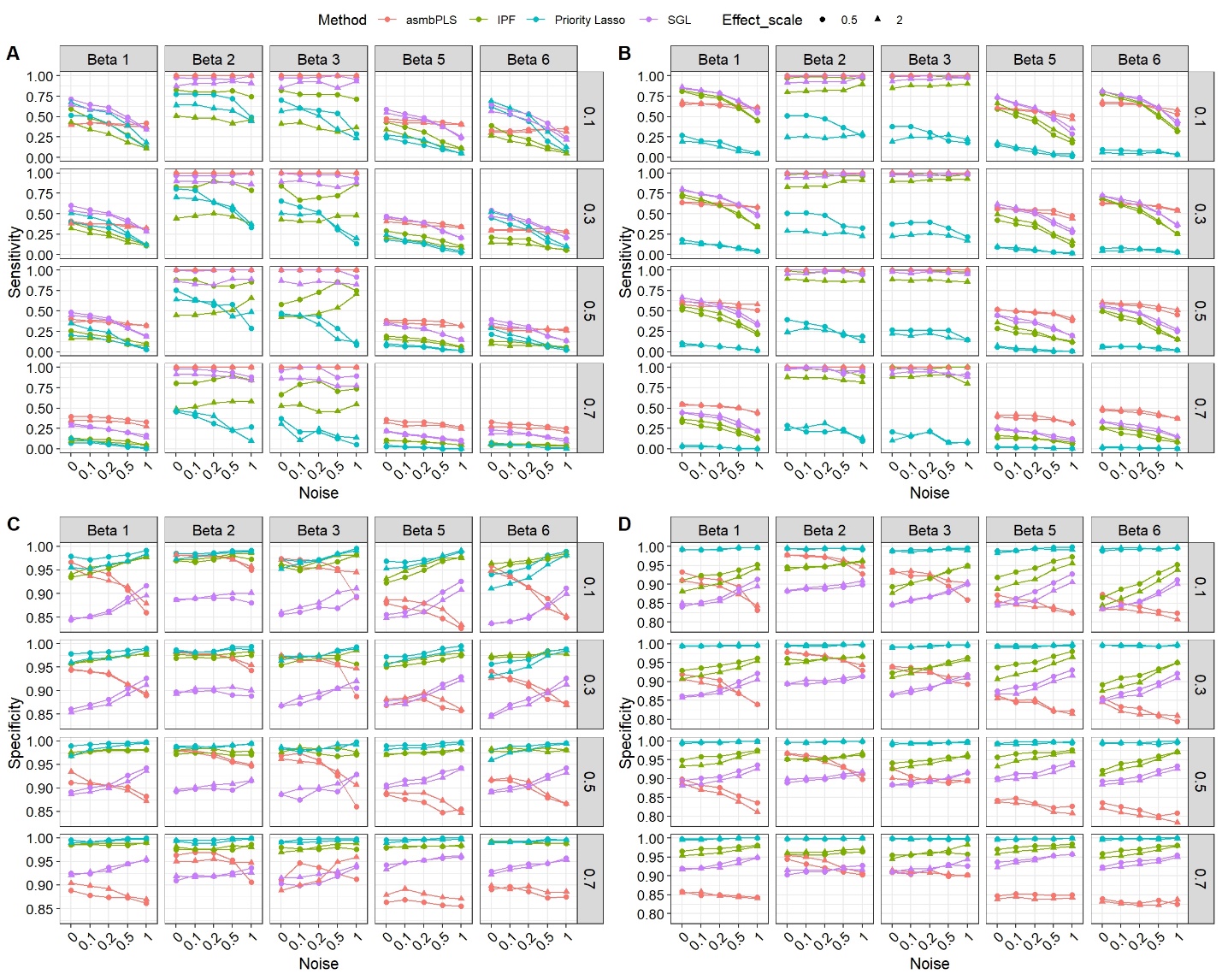
**

**Figure S12.** Sensitivity and specificity of the feature selection for low dimension setting with Weibull distributed survival time. **(A)** Sensitivity for microbiome block; **(B)** Sensitivity for metabolome block; **(C)** Specificity for microbiome block; **(D)** Specificity for metabolome block.

**
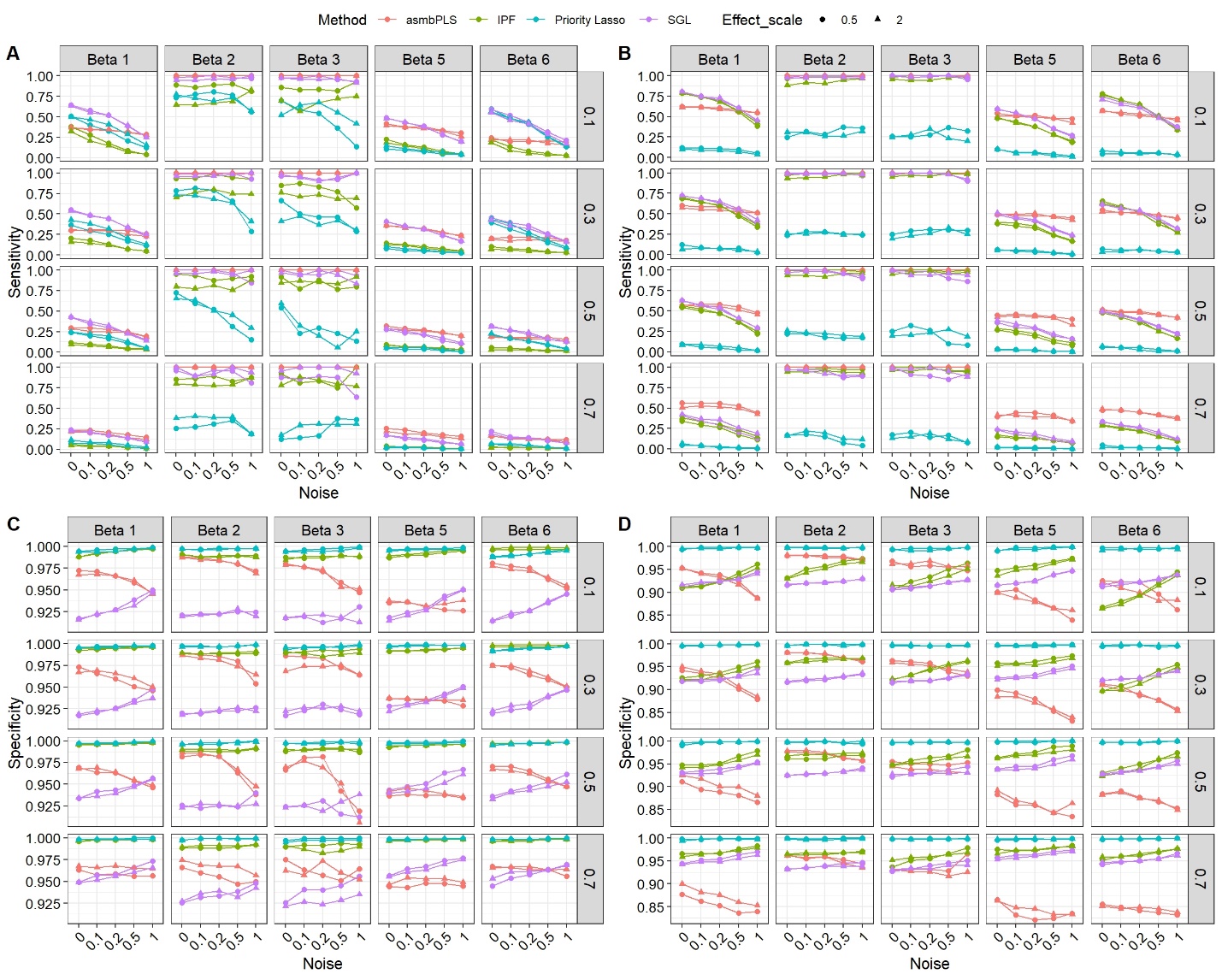
**

**Figure S13.** Sensitivity and specificity of the feature selection for mixed dimension setting with lognormal distributed survival time. **(A)** Sensitivity for microbiome block; **(B)** Sensitivity for metabolome block; **(C)** Specificity for microbiome block; **(D)** Specificity for metabolome block.

**
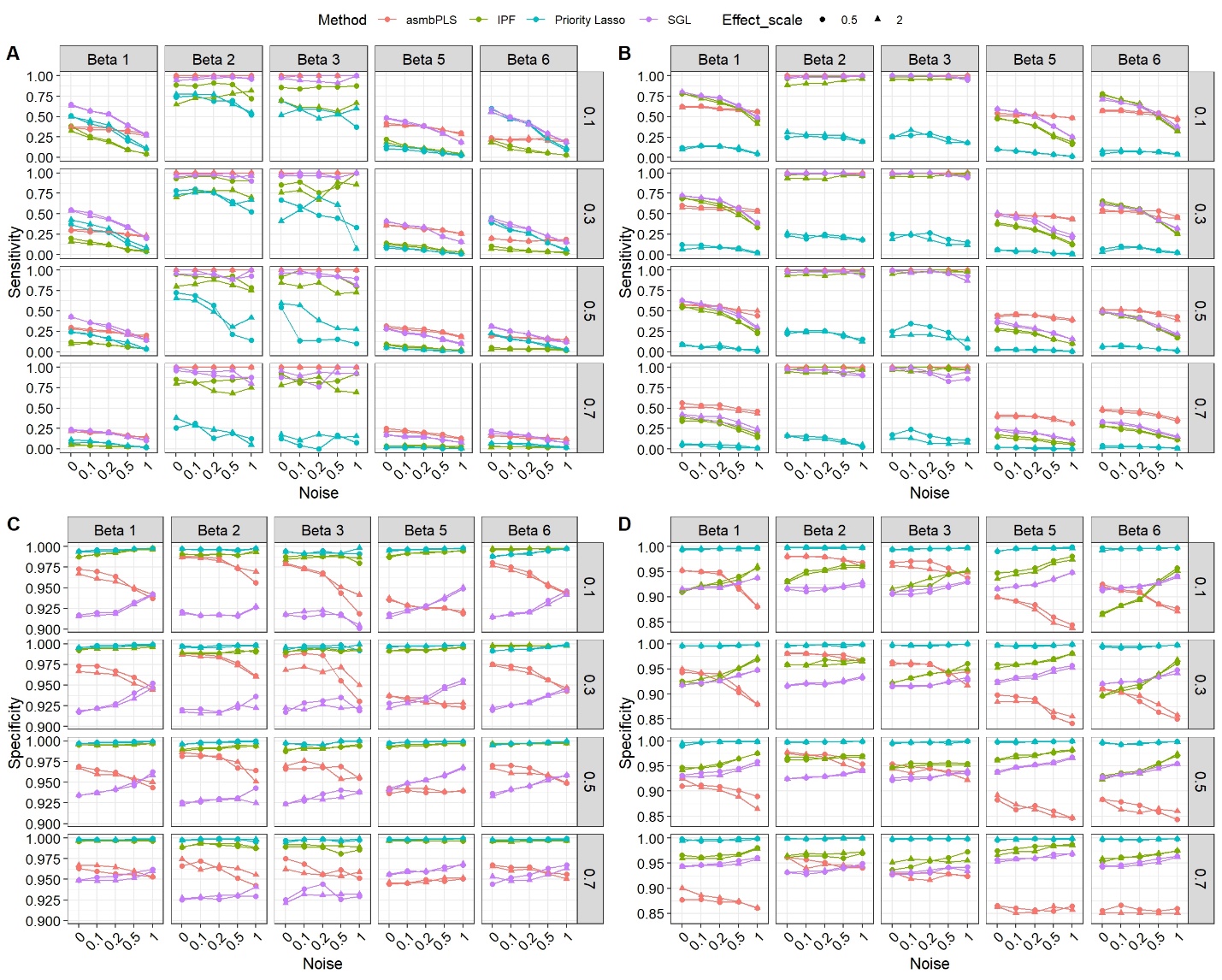
**

**Figure S14.** Sensitivity and specificity of the feature selection for mixed dimension setting with Weibull distributed survival time. **(A)** Sensitivity for microbiome block; **(B)** Sensitivity for metabolome block; **(C)** Specificity for microbiome block; **(D)** Specificity for metabolome block.

**
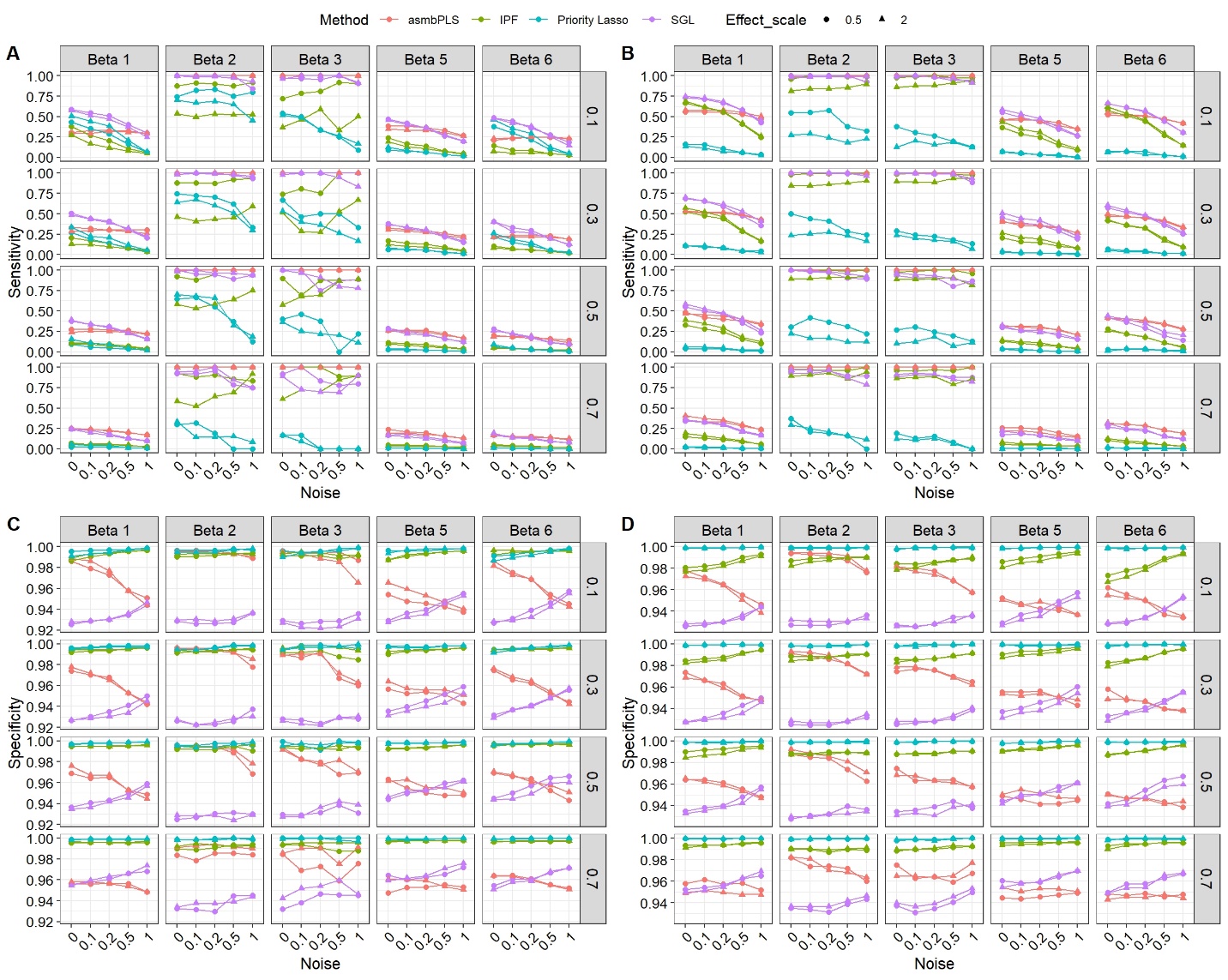
**

**Figure S15.** Sensitivity and specificity of the feature selection for high dimension setting with lognormal distributed survival time. **(A)** Sensitivity for microbiome block; **(B)** Sensitivity for metabolome block; **(C)** Specificity for microbiome block; **(D)** Specificity for metabolome block.

**
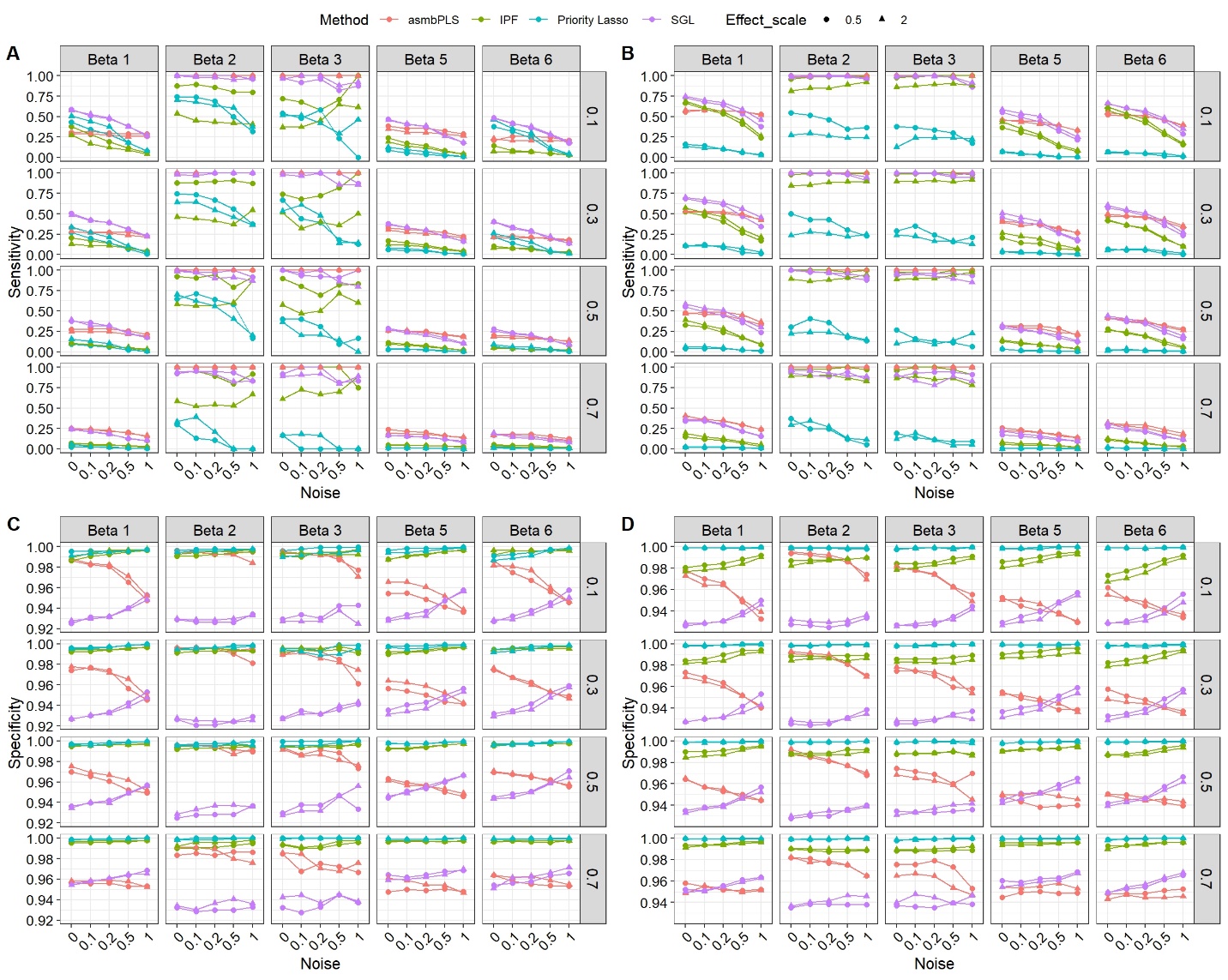
**

**Figure S16.** Sensitivity and specificity of the feature selection for high dimension setting with Weibull distributed survival time. **(A)** Sensitivity for microbiome block; **(B)** Sensitivity for metabolome block; **(C)** Specificity for microbiome block; **(D)** Specificity for metabolome block.
